# Levels and Spatial Patterns of Effective Population Sizes in the Southern Damselfly (*Coenagrion mercuriale*): On the Need to Carefully Interpret Single‐Point and Temporal Estimations to Set Conservation Guidelines

**DOI:** 10.1111/eva.70062

**Published:** 2024-12-24

**Authors:** Agathe Lévêque, Anne Duputié, Vincent Vignon, Fabien Duez, Cécile Godé, Clément Mazoyer, Jean‐François Arnaud

**Affiliations:** ^1^ Univ. Lille, CNRS, UMR 8198—Evo‐Eco‐Paleo Lille France; ^2^ Office de Génie Écologique (O.G.E.) Strasbourg France; ^3^ ALKIOS Avignon France

**Keywords:** conservation biology, damselfly, effective population size, landscape ecology, spatial and temporal genetic structure, urbanisation

## Abstract

The effective population size (*N*_e_) is a key parameter in conservation and evolutionary biology, reflecting the strength of genetic drift and inbreeding. Although demographic estimations of *N*_e_ are logistically and time‐consuming, genetic methods have become more widely used due to increasing data availability. Nonetheless, accurately estimating *N*_e_ remains challenging, with few studies comparing *N*_e_ estimates across molecular markers types and estimators such as single‐sample methods based on linkage disequilibrium or sibship analyses versus methods based on temporal variance in allele frequencies. This study aims at bridging this gap by analysing single‐sample and temporally spaced populations in the southern damselfly (*Coenagrion mercuriale*), a bioindicator Odonata species of conservation concern found in southwestern Europe's freshwater stream networks. A total of 77 local populations were sampled from a semi‐urbanised area located in eastern France near Strasbourg city, yielding 2842 individuals that were genotyped with microsatellites and 958 of which were also genotyped for 2092 SNPs. Spatial genetic structure was stable over time, suggesting porosity between alternate‐year cohorts. When accounting for spatial genetic structure, single‐sample and temporal estimations of *N*_e_ were consistent for each set of molecular markers. Biologically meaningful results were obtained when the effect of migration was minimising by considering metapopulation *N*_e_ estimates based on the level of genetic differentiation and population boundaries. In terms of applied conservation and management, most depicted metapopulations displayed large *N*_e_, indicating no immediate need for conservation measures to mitigate anthropogenic pressures, provided that a continuous suitable freshwater network is maintained. However, urbanisation negatively impacted *N*_e_ levels in populations close to Strasbourg city. Because *N*_e_ is used to inform conservation decisions, caution is crucial in interpreting *N*_e_ estimates, especially in continuously distributed populations undergoing migration. Altogether, our study highlights the challenge of obtaining robust *N*_e_ estimates and the necessity of careful interpretation to set relevant conservation guidelines.

## 1 Introduction

Changes in land use related to human activities can lead to habitat loss and fragmentation, reducing the extent of gene flow among populations and decreasing their census sizes (Fahrig 2003; Wilson et al. 2016). Because of the greater magnitude of genetic drift and the cost of inbreeding, small isolated populations face a substantial risk of decreasing genetic variability over time (Allendorf et al. 2022; Ellegren and Galtier 2016; Frankham, Briscoe, and Ballou 2013; Frankham et al. 2017). Combined with stochastic environmental processes, the deleterious genetic effects associated with the loss of genetic diversity can lead to a reduction in the adaptive potential of populations, which ultimately can drive to a vortex of extinction (Frankham, Briscoe, and Ballou 2013; Saccheri et al. 1998; Soulé and Mills 1998). Consequently, the measurement of contemporary genetic features of populations is a key tool in monitoring the vulnerability of populations to detrimental genetic changes, and in assessing their adaptative potential and long‐term persistence, which is particularly important for threatened species (Allendorf, Hohenlohe, and Luikart 2010; Allendorf et al. 2022; Hohenlohe, Funk, and Rajora 2021; Holderegger et al. 2019).

The effective population size (*N*_e_) is one of the key concepts and parameters in evolutionary biology and conservation genetics, as it reflects the rate at which genetic variation is lost in a population due to genetic drift and inbreeding (Hare et al. 2011; Nei and Tajima 1981; Wang, Santiago, and Caballero 2016; Waples 2005). This concept is linked to the direct relationship between the rate of genetic changes and the size of an ‘ideal’ Wright‐Fisher finite population that satisfies the assumptions of random mating, constant population size and non‐overlapping generations (Fisher 1930; Wright 1931). However, wild populations rarely conform to this model because various factors can reduce the number of individuals contributing to the next generation, including for instance isolated genetic cohorts, unbalanced sex ratios and differential fitness of males and females. This explains why population sizes in the demographic sense (census size) are often greater than population sizes in the genetic sense (effective size, Waples 2016). The *N*_e_ parameter also provides a better understanding of population dynamics in a given region, for example by identifying source‐sink population structure, which can provide important information on the status of populations for their conservation (Frankham, Briscoe, and Ballou 2013; Allendorf et al. 2022).

In this context, numerous methods have been developed to estimate the effective population size in natural populations. The development of genetic methods for estimating *N*_e_ is an active field of research involving increasingly applied advances in genotyping and software dedicated to accurately estimate this crucial population parameter (Luikart et al. 2010; Wang 2009; Wang, Santiago, and Caballero 2016; Waples 2024). Herein, we will focus on contemporary effective population size estimation, that is, involving current generations or just a few generations in the past, as this time scale is the most relevant for conservation genetics purposes (Hare et al. 2011; Luikart et al. 2010; Ryman, Laikre, and Hössjer 2019). Single‐sample estimates assess the *N*_e_ in the generation preceding the sample by measuring the genetic result of processes acting in the parental generation from which the sample is drawn (Hare et al. 2011; Waples 2005). They can be measured from genetic patterns of heterozygosity (Pudovkin, Zaykin, and Hedgecock 1996), linkage disequilibrium (LD, Hill 1981) or individual relatedness (Wang 2009). These methods require just a single sample of multilocus genotypes, making them useful because one does not need to survey several generations. In contrast, the temporal method estimates the harmonic mean of *N*_e_ based on the measurement of the temporal variation of allele frequencies across two or more samples separated in time (Hare et al. 2011; Jorde and Ryman 2007; Nei and Tajima 1981; Pollak 1983; Waples 1989). Compared with other *N*_e_ estimation approaches, the temporal approach makes fewer assumptions and is more robust to some complications in real natural populations, such as population structure or overlapping generations, but is most accurate when the sampling points are separated by many generations (Wang, Santiago, and Caballero 2016).

Yet, a robust estimation of the effective population size and its practical application remain a challenge, particularly for large populations integrated into a metapopulation system with gene flow occurring among neighbouring populations, requiring spatial and temporal considerations to be taken into account in the estimation of this key population parameter (Clarke et al. 2024; Hare et al. 2011; Neel et al. 2013; Waples 2005, 2024). For instance, lumping individuals coming from multiple genetic neighbourhoods generate mixture LD that yields false inferences of *N*_e_ estimates (Kardos and Waples 2024; Neel et al. 2013; Waples 2024). The same holds for temporal approaches for which gene flow may induce bias in *N*_e_ estimates, depending on the magnitude and continuity of gene flow events at a metapopulation scale (Gilbert and Whitlock 2015; Palstra and Ruzzante 2008). In addition, each method for estimating *N*_e_ is subject to numerous assumptions that are often violated in natural populations (Waples 2016). However, to some extent, the precision and accuracy of large *N*_e_ estimates can be improved by increasing sampling efforts for both individuals and loci (Luikart et al. 2010; Wang 2009; Waples 1989).

In this respect, the development of new sequencing technologies (next‐generation sequencing [NGS]) offers the possibility of genotyping thousands of genomic markers (see Bernatchez et al. 2024). SNPs provide a genome‐wide coverage, offering the promise of more accurate estimates of genetic and population demographic parameters important for natural population conservation (Allendorf 2017; Hollenbeck, Portnoy, and Gold 2016; Lehnert et al. 2019; see however Waples, Waples, and Ward 2022). In line with NGS, numerous advances in theory and computational analysis have also considerably improved the estimation of *N*_e_ (Do et al. 2014; Hollenbeck, Portnoy, and Gold 2016; Waples 2024; Waples, Antao, and Luikart 2014; Waples et al. 2018; Waples and Lindley 2018; Zhou et al. 2018). Population genomics approaches have already been used to estimate *N*_e_ (e.g., Browning and Browning 2015; Sovic et al. 2019). Nonetheless, very few studies compared the relative power and biological relevance of single‐sample versus temporal estimates of *N*_e_ using different kinds of molecular markers in natural populations (see however Gargiulo et al. 2023; Morin et al. 2012; Sovic et al. 2019). Our study is thus devoted to bridge this gap by analysing the *N*_e_ of a set of populations of the southern damselfly (*Coenagrion mercuriale*), a protected species, using both single‐sampled populations and temporally spaced populations, and using two different types of molecular markers: classical microsatellite loci and newly developed SNPs.

Therefore, this study aimed at finely characterising the effective population size of a large number of southern damselfly populations located in eastern France. The southern damselfly (*Coenagrion mercuriale*) is an Odonata species that typically inhabits clear, small streams characterised by the occurrence of helophytes. This species is commonly found in southwestern Europe and sparsely encountered in central Europe (Figure S1A). This Odonata species is of high conservation concern because of population declines linked to changes in agricultural practices, particularly at the eastern edge of its range, where the species has gone extinct or is currently close to extinction in seven countries such as Belgium, the Netherlands and Austria (Grand 1996). In eastern France, which is the focus of this study, flying adults gradually emerge from May to August. Southern damselfly adults live for about 2 weeks and mate, and females lay their eggs in the hollow stems of helophytes. Larvae hatch and develop as swimming organisms for 1, 2 or even 3 years before metamorphosing into adults. At its northern limit, in the UK and most central European populations, the southern damselfly is semi‐voltine (i.e., it completes a full generation in 2 years; Purse and Thompson 2002; Sternberg, Buchwald, and Röske 1999), whereas in North Africa it is both univoltine and bi‐voltine (Mahdjoub et al. 2014, 2015). Given this latitudinal gradient of the species' voltinism, we expected the southern damselfly populations located in eastern France to be semi‐voltine. In France, the southern damselfly is widely distributed and locally abundant except for the northernmost region (Figure S1B). In eastern France (Alsace region), the species is widely distributed, although this region is subject to substantial anthropogenic pressures such as strong urbanisation effects owing to the occurrence of Strasbourg Eurométropolis, intensive cereal cultivation and grapevines on the slopes of the Vosges mountains (Figure S1C). Several studies have been carried out to estimate the population census size of this species in different regions (Beaune and Sellier 2021; Couvreur et al. 2008; La Porta and Goretti 2019; Rouquette and Thompson 2005; Thompson and Watts 2006). Nonetheless, only one study was conducted to estimate the *N*_e_ in southern damselfly using genetic tools, highlighting contrasting patterns between demographic and genetic estimates of population size in a single large southern damselfly population located in the UK (Watts, Saccheri, et al. 2007). Demographic estimates of effective population size indeed showed levels of effective size that were overestimated compared with genetic approaches, presumably because of a weak spatial genetic structure and biased estimations of individual reproductive success variance (Watts, Saccheri, et al. 2007).

In addition, although Odonata species are often considered to be efficient fliers, potentially able to disperse over large spatial distances, the southern damselfly has a very low dispersal capability. Mark–release–recapture studies have documented infrequent long‐distance movements of southern damselflies over more than 1 km, most individuals moving less than 50 m (Purse et al. 2003; Watts, Rouquette, et al. 2004; Watts, Rousset, et al. 2007). Adult damselflies are therefore highly sedentary, with only a low frequency (3.4% of marked individuals) of interpatch movements that occur predominantly among neighbouring sites within continuous habitats. Consequently, fine‐scale genetic clustering and isolation by distance both within‐ and between habitat patches can be observed, and spatial patterns of genetic structuring matched closely what could be expected from direct observations of individual movements (Lévêque, Duputié, et al. 2024; Watts, Rouquette, et al. 2004).

The southern damselfly life‐history traits thus provide a unique opportunity and an interesting playground for studying the effective population size, because they can present both geographic and temporal population structures. Indeed, any factor that creates variation in developmental timing can divide a population into discrete cohorts, characterised by their own dynamics, eventually leading to independent demographic and evolutionary trajectories (Battisti, Boato, and Masutti 2000; Bouaouina et al. 2023; Gradish et al. 2019; Wolf and Zwick 1989). By contrast, the possibility of ‘temporal migration’, in which larvae delay metamorphosis for a year and recruit to another cohort, can homogenise different cohorts that would otherwise be independent of each other, as it was shown by Watts and Thompson (2012) in southern damselfly populations located to the north of the species' range (UK).

Here, we used two independent genetic datasets, microsatellites and SNPs, to make conservation‐relevant inferences about genetic characteristics in a large set of southern damselfly populations located in the Alsace region, eastern France. This area is characterised by a variety of agricultural landscapes and the occurrence of a large city, Strasbourg. To go beyond punctual spatial genetic studies, we performed a temporal survey to get further insights into the *N*_e_ of interconnected populations of the southern damselfly where we have previously shown, using microsatellite loci, a low genetic divergence as compared to populations located at the periphery of the species' geographic distribution (Lévêque, Duputié, et al. 2024). The spatial genetic structure observed from microsatellite data in the Alsace region depicted (i) only moderate levels of genetic differentiation, probably because of low resolution of these markers; (ii) a negative impact of urbanisation on the levels of genetic diversity and of gene flow for populations located in the direct vicinity of Strasbourg city and (iii) joint effects of isolation by distance occurring overland and local departures of migration–drift equilibrium (Lévêque, Duputié, et al. 2024). By taking into account the spatial genetic structure, our specific goals were the following: (i) evaluate whether alternate‐year cohorts were genetically distinct with independent evolutionary trajectories or whether gene flow between cohorts ensured a certain degree of genetic cohesion between the sampled years; (ii) estimate and compare contemporary local and metapopulation effective population sizes with both single‐sample and temporal methods for two datasets consisting of microsatellite and SNP markers, respectively; and (iii) identify potential biological and demographic processes affecting the level of estimated *N*_*e*_, such as the occurrence of non‐negligible migration events among nearby populations and the occurrence of a highly urbanised area on the border of the city of Strasbourg.

## 2 Materials and Methods

### 2.1 Study Sites and Sampled Populations

**FIGURE 1:**
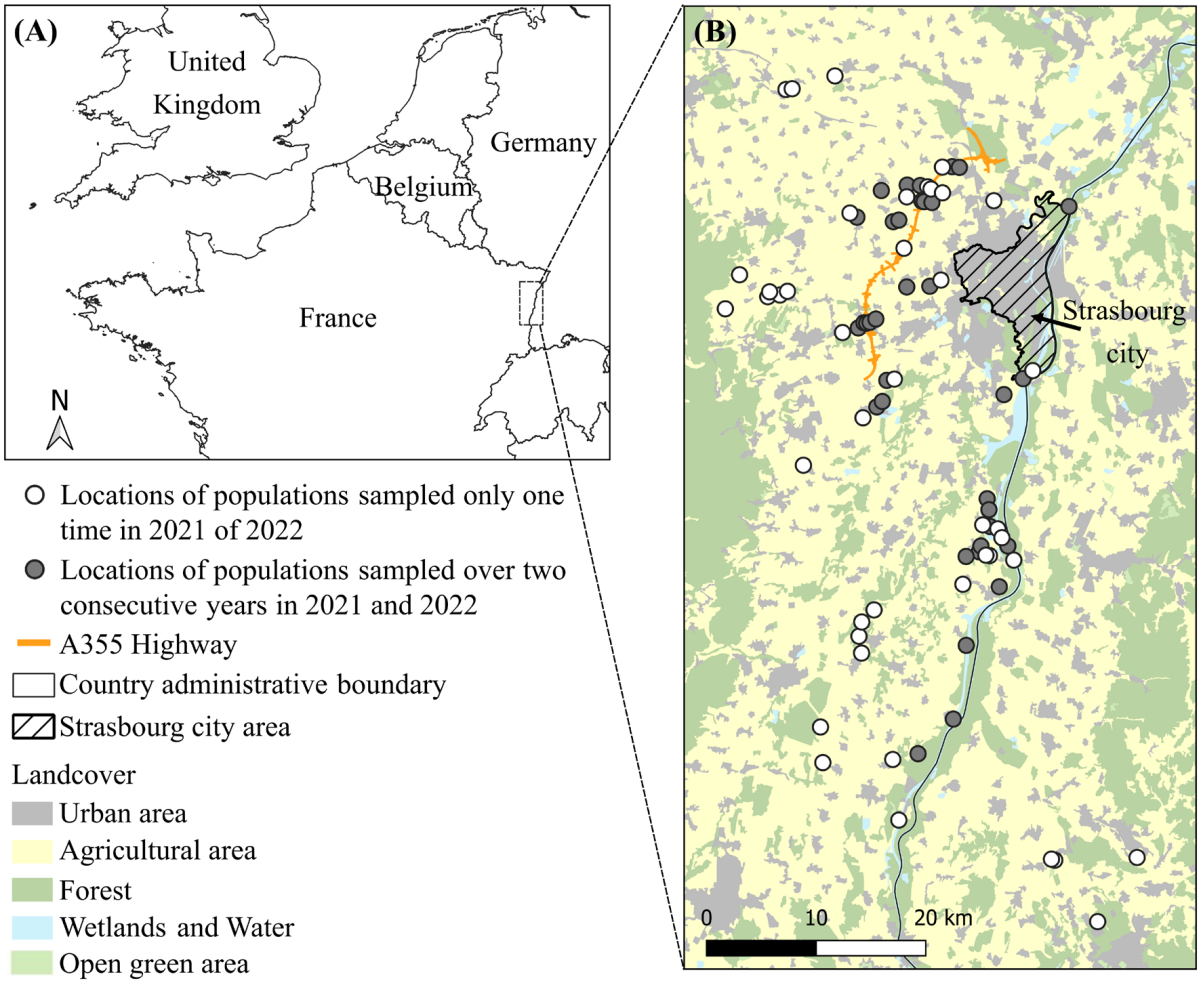
Location of 77 sampled populations of the southern damselfly (*Coenagrion mercuriale*) in northeastern France. Each dot represents one population, characterised by a single sampling in 2021 or 2022 (*N* = 43, white dots) or by two successive samplings in 2021 and 2022, allowing for effective population size (*N*_e_) estimations based on temporal variance in allele frequencies (*N* = 34, dark grey dots). Land cover was simplified from Corine Land Cover Edition 2018. Maps were produced using the open‐source software QGIS v.3.18 (Quantum GIS Development Team).

We collected a total of 2842 southern damselflies from 77 populations in northeastern France, Alsace region (Figure 1). Adult individuals were collected using an insect net in late spring (May to July) in 2021 and 2022. Thirty‐four populations were sampled in both years (Table S1). Purse et al. (2003) showed that mature adults disperse over very short distances (on average < 25 m) even within continuous suitable habitats, with a low emigration rate ranging from 1.3% to 11.4% among immediate neighbouring habitat patches (see also Watts, Rouquette, et al. 2004). Therefore, we defined local populations as a group of randomly sampled adults within a specific geographic location, spanning a 200‐m transect along watercourses, and separated by at least 500 m. Minimum sampling size was 10 individuals to allow biologically relevant estimation of effective population sizes: 10–39 individuals were collected per population (mean sampling size 25.6, s.d. 6.6). Geographic coordinates and sampling sizes of each population can be found in Table S1. Two kinds of samples were collected: whole individual body or only the right middle leg of each individual, the latter method being non‐lethal and not impacting the damselfly's survival (Fincke and Hadrys 2001). All samples were stored in 100% ethanol until DNA extraction.

### 2.2 Genotyping Using Microsatellite and SNP Loci

Individuals were genotyped using two different kinds of molecular markers: (i) one based on 10 microsatellite loci previously described by Watts, Wu, et al. (2004), Watts, Thompson, and Kemp (2004) and using the full dataset of 2842 sampled individuals (77 populations, 34 of which being sampled in both years); (ii) the other one using a set of 2092 SNPs on a subset of 958 individuals whose full body was available to ensure a sufficient quantity of DNA material, representing 56 populations, 11 of which were sampled in both years (Table S1). Both microsatellites and SNPs were used to estimate contemporary effective population sizes.

For whole individuals, we removed abdomens from the rest of the body to avoid sampling intestinal microbiota, and we crushed all samples using MN Beads Type D (Macherey‐Nagel) as described in Lévêque, Duputié et al. (2024). We extracted total genomic DNA from each whole individual sample using NucleoMag Tissue Kit (Macherey‐Nagel) according to the manufacturer's recommendations. For leg samples, we purified DNA with 12 μL NucleoMag B‐beads and 12 μL of pure water and eluted in 50 μL.

We genotyped all samples using 10 unlinked nuclear microsatellite loci named LIST002, LIST023, LIST034, LIST035, LIST037, LIST042, LIST062, LIST024, LIST063, and LIST066, isolated and described in Watts, Wu, et al. (2004), Watts, Thompson, and Kemp (2004) and following the protocol detailed in Lévêque, Duputié et al. (2024).

We also genotyped southern damselfly individuals using 2092 SNPs generated using the Tecan Genomics' Allegro Targeted Genotyping (ATG, Redwood City, United States) method. All details of the library preparation and bioinformatics analysis are detailed in Lévêque, Arnaud, et al. (2024). Briefly, based on RADloci constructed from a ddRAD library, 12,000 SPET probes were designed to recapture 6000 SNPs. Target genomic libraries were prepared following the manufacturer guidelines for preparing the SPET libraries and were sequenced on a MiSeq System (1 × 150 bp, Illumina Inc. San Diego, CA, USA) at the GenoScreen sequencing platform (Lille, France). The following bioinformatics analyses included sequences' cleaning, alignment to the reference (*bowtie2*; Langmead and Salzberg 2012), variant calling following the software best practices for germline short‐variant discovery implemented in GATK Best Practices and filtering to obtain reliable SNPs. Challenges encountered mostly included library amplification failure with Allegro i5 indexes, sequence recapture failure and coverage depth discrepancies. After removing loci showing aberrant *F*_IS_ estimates (441 loci) and loci under selection (8 loci), we obtained genotypes for 2092 biallelic SNPs, each located on a different RADcontig to avoid pseudoreplication due to LD (see Lévêque, Arnaud, et al. 2024). All other initially targeted SNPs were removed because of technical failures of single primer enrichment technology (ATG method).

### 2.3 Temporal Genetic Differentiation

First, we assessed whether the population genetic structure was stable over years. We investigated this issue using only the microsatellite loci because of more resampled populations genotyped using this kind of molecular marker. We computed pairwise estimates of genetic differentiation (*F*_ST_) between all pairs of samples (between cohorts and between spatial locations) for populations sampled in both years. We tested the statistical significance of pairwise *F*_ST_ using permutation tests (10,000 multilocus genotype permutations among both temporal samples and spatial locations) implemented in fstat v.2.9.4 (Goudet 2003). To assess whether the spatial genetic structure was stable over cohorts, we regressed pairwise *F*_ST_ values between equivalent pairs of populations from each cohort and tested for statistical significance using a Mantel statistic *rz* (Smouse, Long, and Sokal 1986) with the *mantel.rtest* function of the R package *ade4* with 10,000 permutations (Dray and Dufour 2007).

Second, we evaluated the importance of the sampling year on genetic differentiation by calculating hierarchical *F*‐statistics (Yang 1998). We did not use statistics based on variance in microsatellite allele length and assuming a stepwise mutation model (SMM) because whereas SMM‐based statistics may be well suited for highly divergent populations in a large biogeographical context, differentiation statistics based on genetic drift, as estimated from microsatellite allele frequencies, like *F‐*statistics, are the most valuable tools for studying moderately genetically structured populations (Arnaud 2003; Balloux and Lugon‐Moulin 2002). We constructed a two‐level hierarchy, using years of sampling as the outermost level and the sampling site as the innermost level. We estimated the variance associated with each level using the function *varcomp.glob* in the R package *hierfstat* v.0.5‐11 (Goudet 2005). We assessed the statistical significance of variance explained by each level using the permutation algorithms with the functions *test.between* (year of sampling level) and *test.within* (population level) implemented in the same package. As above, we only carried out this analysis using the microsatellite dataset, as it contained more populations sampled over 2 years compared to the SNP dataset (34 populations and 11 populations, respectively). Besides, it allowed a comparison with previous results obtained for this species (see Watts and Thompson 2012).

### 2.4 Spatial Genetic Differentiation and Population Genetic Affiliations

Using microsatellite loci, Lévêque, Duputié, et al. (2024) showed low levels of genetic differentiation and isolation by distance over the studied area, with a mean multilocus *F*_ST_ estimate of 0.022. Thus, each sampling site could not be considered as an independent isolated population, but may instead be part of a larger metapopulation whose local elements are connected by some level of gene flow (Kardos and Waples 2024; Neel et al. 2013; Palstra and Ruzzante 2008; Waples 2024). Ignoring such fine‐scaled population structure may affect inferences of contemporary local *N*_e_ estimations in contrasting ways, depending on whether the migrants are genetically divergent or not, and on whether the migration rate is > 5%–10% (Kardos and Waples 2024; Waples 2024; Waples and England 2011). Thus, to disentangle local effective population size *N*_e_ from metapopulation *N*_e_, we used two complementary approaches to identify genetic groups: (i) we identified groups of populations that showed a lack of significant pairwise genetic differentiation and (ii) we also applied Bayesian clustering methods to identify genetic boundaries, which also enabled further assessment of potential migrant individuals.

First, we calculated pairwise *F*_ST_ estimates for all populations in both years of sampling with the newly developed and more resolutive SNP markers described in Lévêque, Arnaud, et al. (2024) and using the R package *hierfstat* v.0.5‐11 (Goudet 2005). Each pairwise comparison was tested for statistical significance using 1000 bootstrap replicates. Keeping in mind that grouping individuals from diverse genetic neighbourhoods across space and time to create a single sample may either increase or decrease the *N*_e_ estimates, depending on the genetic background of individuals (see Kardos and Waples 2024; Neel et al. 2013; Waples and England 2011), all populations not significantly genetically differentiated using the SNP dataset were then grouped to further get estimates of metapopulation *N*_e_, called hereafter metapopulation *N*_e_–*F*_ST_.

Then, to assess population genetic delineations and to control for recent migration events that could bias *N*_e_ estimation, we analysed both microsatellite and SNP datasets using Bayesian clustering methods. The microsatellite dataset was analysed using structure v.2.3.3 (Pritchard, Stephens, and Donnelly 2000) with the admixture model. We ran the analyses using of 500,000 burn‐in Markov Chain Monte Carlo (MCMC), followed by 5,000,000 MCMC iterations performed on 15 replicates for each *K* number of clusters tested, ranging from 1 to 20. The SNP dataset was analysed using faststructure v.1.0 (Raj, Stephens, and Pritchard 2014) with default settings, and we also tested *K* values ranging from 1 to 20 with 15 replicates and random seeds for each *K* value. For both molecular datasets, we identified the most likely value for *K* to depict population affiliation among all samples using the Δ*K* statistic (Evanno, Regnaut, and Goudet 2005) with structureselector (Li and Liu 2018) and the R package *pophelper* v.2.3.1. A drawback of population clustering analyses is their tendency to focus on the broadest genetic subdivisions, potentially masking finer scale genetic patterns (Evanno, Regnaut, and Goudet 2005; McCartney‐Melstad, Gidiş, and Bradley Shaffer 2018; Vähä et al. 2007). Therefore, to ensure that finer, hierarchical levels of population structure were clearly identified, samples were reanalysed separately into the main groups depicted by first population assignments, testing *K* values from 1 to 6 with 15 replicates. Using the population clusters identified by Bayesian clustering, we then used these population groupings to estimate metapopulation *N*_e_, hereafter referred to as metapopulation *N*_e_‐Bayesian clustering.

### 2.5 Estimation of Effective Population Size

We estimated contemporary effective population sizes for both datasets using two single‐sample estimators and three temporal (two‐sample) estimators. First, we estimated single‐sample contemporary *N*_e_ using the method based on LD (Hill 1981; Waples 2006; Waples and Do 2010) and implemented in the program neestimator v.2.1 (Do et al. 2014). This analysis was conducted assuming random mating, using 0.05 as a threshold for the lowest allele frequency and using the jackknife‐based confidence intervals. Because our SNP dataset contained loci with a minor allele frequency as low as 1%, this reduced the number of loci used in the calculation, but at least 1200 loci remained in each population (see Figure S2). Second, we applied a maximum likelihood method to estimate single‐sample contemporary *N*_e_. To this end, we used the COLONY software v.2.0.7.0 (Jones and Wang 2010) and performed a full‐likelihood analysis method dedicated to dioecious diploid species, with medium‐length runs, assuming random mating and polygamy for both males and females and with no sibship prior. All other parameters were set as default. This maximum likelihood method estimates *N*_e_ based on inferred sibship frequencies among samples, with associated confidence intervals obtained through bootstrap resampling (Wang 2009). Third, *N*_e_ were estimated for temporally resampled populations using a method based on temporal changes in allele frequencies (see Waples 1989) and three different estimators for computing the standardised variance in allele frequency *F*: *F*_e_ (Nei and Tajima 1981), *F*_k_ (Pollak 1983) and *F*_s_ (Jorde and Ryman 2007). As in Gargiulo et al. (2023), to evaluate the influence of migration on metapopulation *N*_e_‐Bayesian clustering estimations, we removed the potential migrants (i.e., individuals displaying a proportion of admixture > 20%) and re‐estimated *N*_e_ for each identified genetic cluster.

## 3 Results

### 3.1 Levels of Genetic Differentiation Between Cohorts

**FIGURE 2:**
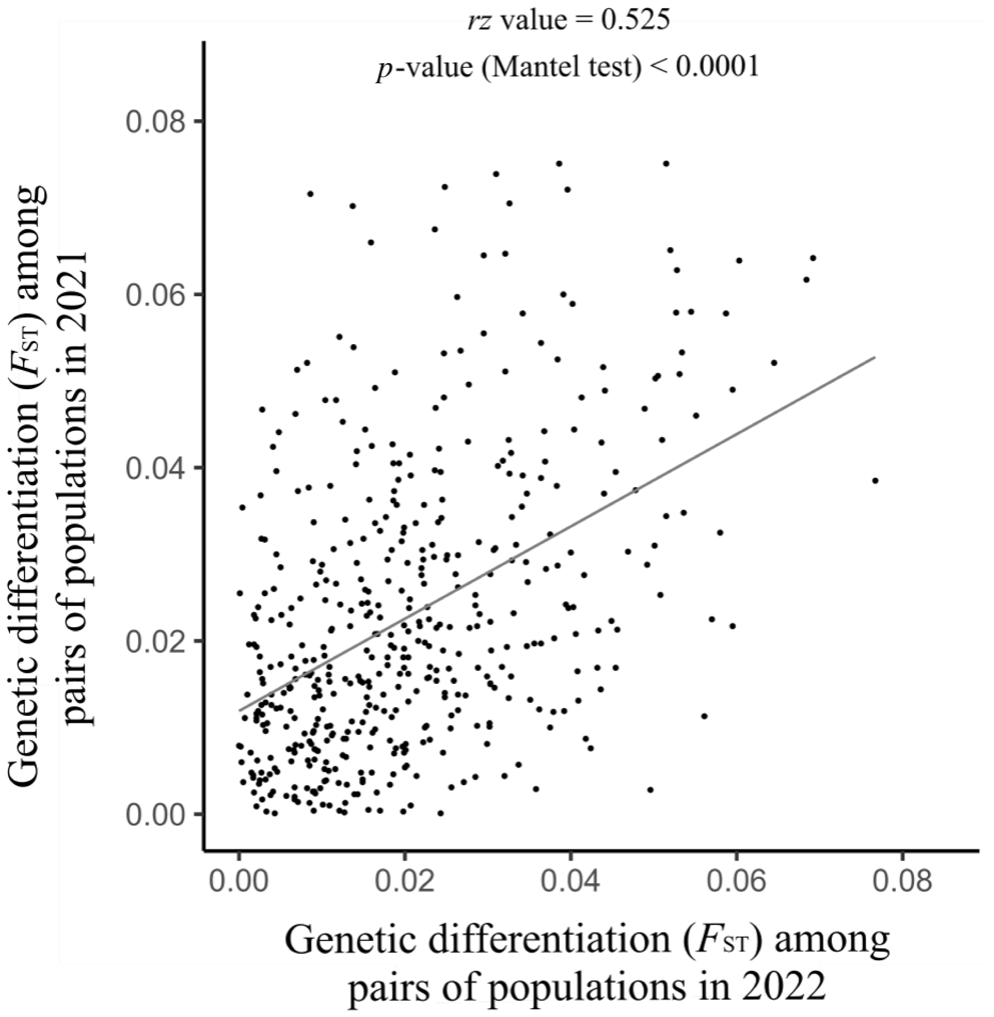
Scatterplot of estimates of pairwise genetic differentiation (*F*_ST_) for identical pairs of southern damselfly (*Coenagrion mercuriale*) populations sampled in 2021 and in 2022.

Using microsatellite loci, none of the pairwise estimates of genetic differentiation between temporal samples of populations differed from 0 after Bonferroni correction (Table S2). Besides, pairwise values of *F*_ST_ between equivalent pairs of populations from each cohort were significantly correlated, indicating a stable population genetic structure over time (Mantel *rz* = 0.525, *p* < 0.0001; Figure 2). Likewise, the hierarchical *F*‐statistics analysis clearly showed significant levels of genetic differentiation among populations within the same year of sampling (*F*_population/year_ = 0.165; *p* = 0.01), but not among years (*F*_year/total_ = −0.018; *p* > 0.05).

### 3.2 Levels of Spatial Genetic Differentiation

**FIGURE 3:**
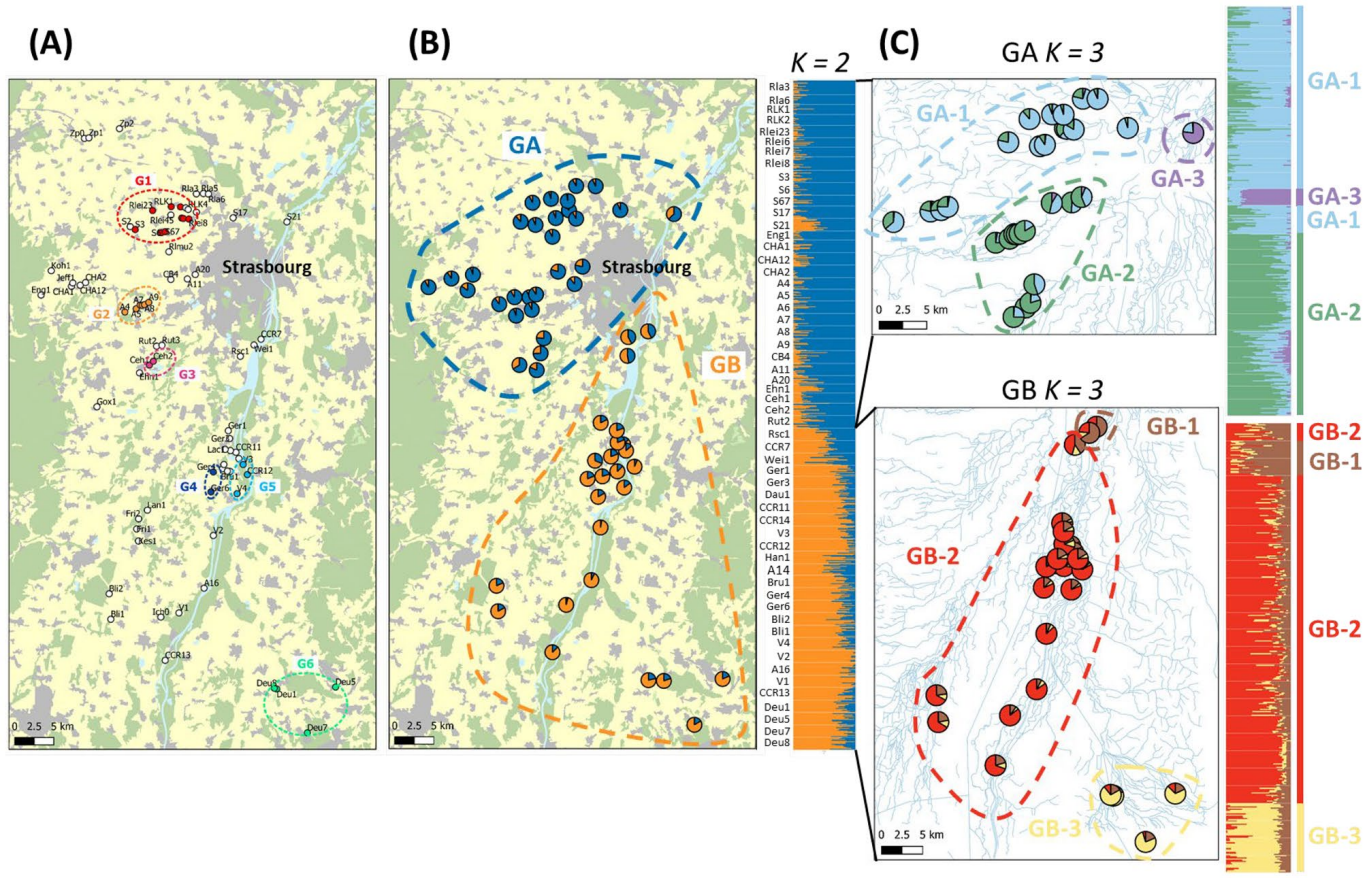
Location of 77 populations of the southern damselfly (*Coenagrion mercuriale*) sampled in northeastern France and the delimitation of population boundaries based on (A) non‐significant pairwise FST estimates (see Figure S3) or (B, C) Bayesian clustering. (A) Populations exhibiting non‐significant pairwise *F*_ST_ estimates were grouped into six different groups of populations, labelled G1, G2, G3, G4, G5 and G6, pictured by coloured dashed ellipses. Populations that were significantly genetically differentiated from the others are represented by white dots. (B) Bayesian clustering results showing a hierarchical genetic structure in southern damselfly populations. Pie charts depict mean population membership probabilities for *K* clusters for each sampling location of southern damselfly population and corresponding bar plots show individual membership probabilities to belong to *K* clusters (each cluster is represented by a different colour, and each horizontal bar represents an individual). Bayesian clustering was initially conducted with all samples (with an optimal *K* = 2), followed by subsequent independent runs within the two main population grouping depicted (C). Based on within‐group assignments, populations were further grouped into six different groups of populations, labelled GA‐1, GA‐2, GA‐S21, GB‐1, GB‐2 and GB‐3. Land cover was simplified from Corine Land Cover Edition 2018. Watercourses from Copernicus Land Monitoring Service, 2019: EU‐Hydro.

With respect to spatial genetic differentiation and using SNP markers, almost all populations were significantly differentiated from each other: *F*_ST_ ranged from −0.005 to 0.057, and out of 1109 comparisons, only 64 were non‐significant and involved only geographically close populations located within the same watercourse network (see Figure S3). Therefore, we pooled individuals from spatially distinct but non‐genetically differentiated populations into six different metapopulations (see Figure 3A). The groups were as follows: Group 1 (G1) included populations RLK1, RLK2, Rlei23, Rlei6, Rlei7, Rlei8, S3, S6 and S67; Group 2 (G2) consisted of populations A4 to A9; Group 3 (G3) composed of populations Ceh1 and Ceh2; Group 4 (G4) comprised populations Ger4 and Ger6; Group 5 (G5) contained populations V3, V4 and CCR12 and finally Group 6 (G6) gathered populations Deu1, Deu5, Deu7 and Deu 8. Based on this grouping, six additional metapopulation *N*_e_, named ‘metapopulation *N*_e_–*F*_ST_’, were calculated using single‐sampled and temporally spaced estimators with both microsatellite and SNP loci (see below).

### 3.3 Depiction of Population Boundaries

A clear genetic partitioning occurred over the whole dataset of local sampled populations, with the Δ*K* statistics indicating a best fit at *K* = 2 using either microsatellite or SNP datasets (data not shown). The resulting two genetic clusters made sense from a geographical point of view and to a lesser extent from a hydrological point of view, and corresponded to populations located in the northwestern (GA) and the southeastern parts of the city of Strasbourg (GB, see Figures 3B and S4). Subsequent faststructure runs using the SNP dataset within each of these main genetic groups revealed sub‐structuring, with the most likely number of populations being *K* = 3 for both the GA and GB main groups (Figure 3C). Within the GA grouping, a first group encompassed populations located along a hydrographic network to the northwest of Strasbourg city and additional populations located farther west (GA‐1). A second group included populations located along two river systems to the west of the city (GA‐2). Finally, the S21 population located to the northeast of Strasbourg city clearly behaved as a distinct genetic entity (GA‐3). Within the GB region, three distinct genetic groups were also identified. A first genetic group corresponded to the populations found south, near the city of Strasbourg (GB‐1 composed of the CCR7 and Wei1 populations). A second group included all populations located within the hydrographic networks south of Strasbourg (GB‐2). Finally, a third genetic cluster comprised the populations located in Germany (GB‐3). Based on this Bayesian clustering, five additional metapopulation *N*_e_ estimates (named Metapopulation *N*_e_‐Bayesian clustering) were calculated using single‐sample and temporal estimators for both microsatellite and SNP datasets (see below). Cluster GA‐3 was not included in these additional analyses as it corresponded to local *N*_e_ estimates already estimated for the S21 population. Population names associated with each metapopulation can be found in Table S3.

### 3.4 Estimates of Effective Population Sizes

#### 3.4.1 Local Effective Population Size

Local effective population size estimates varied depending on the type of genetic data and the method used to generate *N*_e_ estimates (Figures S5–S7). Altogether, local *N*_e_ estimates were consistent between estimators for the same type of genetic marker, although notable differences emerged between estimates derived from microsatellite versus SNP markers (see Figure S5). Full details of local *N*_e_ estimations generated using the different methods are presented in Tables S4 and S5, and can be visualised in a density plot displayed in Figure S5. For single‐sample estimators, a large proportion of populations were estimated to have an infinite local *N*_e_, with 31 out of 77 populations for the LD estimates using microsatellite data, and 23 and 36 out of 56 populations using SNPs markers for LD and sibship‐based estimates, respectively (see Figure S6 and Table S4). Finite local *N*_e_ estimates from LD ranged from 11.5 to over 3500 using the microsatellite dataset and from 41.5 to over 21,200 for the SNP dataset (Table S4, Figure S6). Local *N*_e_ estimates using the sibship estimator ranged between 19 and 44 and between 52 and 420 for microsatellites and SNPs, respectively. Contrasting with all other single‐sample local *N*_e_ estimates, the sibship estimates using microsatellite data yielded much lower *N*_e_ estimates, all centered around 30 and with no infinite estimates of local *N*_e_ (Figure S5).

Temporal local *N*_e_ estimates calculated with the three estimators were extremely similar when using microsatellite data (Figure S5). However, using SNP data, there were some differences between estimators, particularly in the estimates produced by the Nei and Tajima (1981) estimator. Once again, a high number of populations were estimated as infinite in size using microsatellite data, ranging from 14 to 17 populations out of 34, depending on estimators. Using SNPs, these proportions were lower for Pollak (1983) and Jorde and Ryman (2007) *N*_e_ estimators (0 and 2 out of 11 populations, respectively). Finite local *N*_e_ estimates from Pollak (1983) ranged from 12 to over 1000 using the microsatellite dataset, and from 43 to over 4000 for the SNP dataset (Table S5, Figure S7). *N*_e_ estimates using Nei and Tajima (1981) estimator ranged between 14 and 315 and between 80 and 1011 for microsatellites and SNPs, respectively. Finally, local *N*_e_ estimates from Jorde and Ryman (2007) ranged between 10 and 1144 and between 38 and 423 for microsatellites and SNPs, respectively (Table S5, Figure S7). Local population S21, also considered as a unique genetic entity using Bayesian clustering, exhibited the most salient result in terms of conservation management. This population located in the direct vicinity of the Strasbourg city displayed consistently low *N*_e_ estimates among markers types, around 50 individuals using single‐sample estimators and around 13 individuals using temporally spaced sample estimates (Tables S4 and S5).

#### 3.4.2 Metapopulation *N*_e_– *F*_ST_

When focusing on metapopulation *N*_e_ based on the grouping of non‐genetically differentiated populations, 5 out of 6 metapopulations were characterised by high (> 1300) or infinite *N*_e_ estimates using the LD method with microsatellites, again in contrast to the sibship estimator that still yielded lower estimates ranging from 31 to 100 (Table 1a). Using the SNP dataset, the LD method gave metapopulation *N*_e_–*F*_ST_ ranging from 238 to 1669 with no infinite estimates, and the sibship estimator yielded higher metapopulation *N*_e_–*F*_ST_ estimates ranging from 468 to infinity (Table 1a). Temporal (two‐sample) methods applied to the microsatellite dataset showed congruent results, with consistently infinite estimates of metapopulation *N*_e_–*F*_ST_ for G3 and G4, the remaining estimates ranging from 79 to 694, depending on the estimator (Table 1b). Using the SNP dataset, the most salient result was a low metapopulation *N*_e_–*F*_ST_ for the G3 grouping, ranging from 56 to 73 depending on the temporal estimator used. Other groupings of populations exhibited more elevated metapopulation *N*_e_–*F*_ST_ ranging from 259 to infinite estimates (Table 1b).

**TABLE 1:** Metapopulation *N*_e_–*F*_ST_ estimates for the two molecular datasets (microsatellites and SNPs), using (a) single‐sample estimates: The linkage disequilibrium method (Hill 1981; Waples 2006; Waples and Do 2010) and the sibship assignment (Wang 2009), and (b) temporal sample estimates, using Pollak's (1983) estimator, Nei and Tajima's (1981) estimator and Jorde and Ryman's (2007) estimator. Values in square brackets represent 95% confidence intervals based on a jackknife procedure.

(a)	Microsatellites					SNPs				
Metapopulation *N*_e_–*F*_ST_	Sample size	LD method		Sibship assignment method		Sample size	LD method		Sibship assignment method	
		Estimated *N*_e_	95% CI	Estimated *N*_e_	95% CI		Estimated *N*_e_	95% CI	Estimated *N*_e_	95% CI
G1	201	1360.6	[169.5; ∞]	100	[76; 132]	122	870	[431; 61835.2]	2460	[1501; 6212]
G2	170	∞	[4402.2; ∞]	82	[61; 113]	88	761.9	[290; ∞]	2552	[1421; 12,764]
G3	40	∞	[70.3; ∞]	30	[18; 52]	27	238.1	[95.9; ∞]	468	[224; ∞]
G4	49	∞	[59.4; ∞]	31	[20; 53]	30	723.6	[207.7; ∞]	870	[362; ∞]
G5	73	∞	[209.8; ∞]	51	[35; 77]	45	839	[212.5; ∞]	1980	[836; ∞]
G6	111	353.6	[84.8; ∞]	61	[44; 87]	60	1669.9	[594.7; ∞]	∞	[1, ∞]

(b)	Sampling sizes				Pollak (1983)				Nei and Tajima (1981)				Jorde and Ryman (2007)			
Metapopulation *N*_e_–*F*_ST_	Microsatellites		SNPs		Microsatellites		SNPs		Microsatellites		SNPs		Microsatellites		SNPs	
	Sample size 2021	Sample size 2022	Sample size 2021	Sample size 2022	Estimated *N*_e_	95% CI	Estimated *N*_e_	95% CI	Estimated *N*_e_	95% CI	Estimated *N*_e_	95% CI	Estimated *N*_e_	95% CI	Estimated *N*_e_	95% CI
G1	201	218	NA	NA	694.1	[92.4; ∞]	NA	NA	444.1	[88.4; ∞]	NA	NA	353.2	[110.1; ∞]	NA	NA
G2	150	149	72	73	68.8	[11.2; ∞]	13,079	[380.6; ∞]	84.2	[19.2; ∞]	∞	[374.8; ∞]	110.4	[53.3; ∞]	938.9	[231.8; ∞]
G3	56	40	23	27	∞	[40.9; ∞]	58	[39.4; 101]	∞	[53.1; ∞]	73.2	[46.3; 153.1]	∞	[144.3; ∞]	56.6	[37.4; 116]
G4	49	30	NA	NA	∞	[27.1; ∞]	NA	NA	381.7	[17.5; ∞]	NA	NA	79.1	[20.4; ∞]	NA	NA
G5	41	99	30	15	∞	[1265.1; ∞]	259.1	[74.4; ∞]	∞	[243.4; ∞]	∞	[109; ∞]	∞	[∞; ∞]	1056.9	[77.7; ∞]

#### 3.4.3 Metapopulation *N*_e_‐Bayesian Clustering

Looking at the single‐sample estimates of metapopulation *N*_e_ based on population groupings after Bayesian clustering (Figure 3C), the three metapopulations GA‐1, GA‐2 and GB‐2 showed high levels of effective population size using the LD method with microsatellites (> 1220), again in contrast to the sibship estimator, which gave lower estimates ranging from 35 to 273 (Table 2a). Using the SNP dataset, four metapopulations GA‐1, GA‐2, GB‐2 and GB‐3 showed high values of *N*_e_, ranging from 771.4 to 1669.9 for the LD estimate, and from 2962 to infinity using the sibship estimator. Metapopulation GB‐1 exhibited low levels of *N*_e_ compared with the others for both types of markers, with *N*_e_ estimates of 78.2 and 150 for the LD estimator and from 150 to 218 for the sibship estimator for microsatellite and SNP markers, respectively. Temporal (two‐sample) methods applied to the microsatellite and the SNP datasets showed lower levels of *N*_e_ compared to those obtained with single‐sample methods (Table 2b). This was particularly the case for the GA‐1 and GB‐1 metapopulations, for which two‐sample estimates of *N*_e_ based on SNP data gave *N*_e_ values ranging between 20 and 38.5 depending on the estimators. Interestingly, all temporal *N*_e_ estimates were consistently higher when using microsatellite loci as compared to SNPs (Table 2b).

**TABLE 2:** Metapopulation *N*_e_‐Bayesian clustering estimates using the two molecular datasets (microsatellites and SNPs), using (a) single‐sample *N*_e_ estimates: the linkage disequilibrium method (Hill 1981; Waples 2006; Waples and Do 2010) and sibship assignment (Wang 2009) and (b) temporal samples, using Pollak's (1983) estimator, Nei and Tajima's (1981) estimator and Jorde and Ryman's (2007) estimator. Values in square brackets represent 95% confidence intervals based on a jackknife procedure.

(a)	Microsatellites					SNPs				
Metapopulation *N*_e_‐Bayesian clustering	Sample size	LD method		Sibship assignment method		Sample size	LD method		Sibship assignment method	
		Estimated *N*_e_	95% CI	Estimated *N*_e_	95% CI		Estimated *N*_e_	95% CI	Estimated *N*_e_	95% CI
GA‐1	599	4317.5	[554.8; ∞]	239	[199; 288]	222	938.8	[659.5; 1650.5]	4673	[3246; 8305]
GA‐2	353	1220.8	[321.4; ∞]	160	[130; 199]	181	771.4	[499.6; 1686.2]	2962	[2026; 5206]
GB‐1	49	78.2	[27.0; ∞]	35	[23; 59]	30	150.0	[59.5; ∞]	218	[127; 582]
GB‐2	709	2266.4	[574.4; ∞]	273	[228; 326]	297	954.0	[796.8; 1220.9]	3822	[2999; 5409]
GB‐3	111	353.6	[84.8; ∞]	70	[51; 99]	60	1669.9	[594.7; ∞]	∞	[1; ∞]

(b)	Sampling sizes				Pollak (1983)				Nei and Tajima (1981)				Jorde and Ryman (2007)			
Metapopulation *N*_e_‐Bayesian clustering	Microsatellites		SNPs		Microsatellites		SNPs		Microsatellites		SNPs		Microsatellites		SNPs	
	Sample size 2021	Sample size 2022	Sample size 2021	Sample size 2022	Estimated *N*_e_	95% CI	Estimated *N*_e_	95% CI	Estimated *N*_e_	95% CI	Estimated *N*_e_	95% CI	Estimated *N*_e_	95% CI	Estimated *N*_e_	95% CI
GA‐1	292	574	166	56	128.0	[31.1; 743.0]	37.1	[32.1; 43.1]	162.5	[43.5; 1109.2]	38.5	[33.2; 44.9]	311.7	[112.8; ∞]	37.7	[32.4; 45.1]
GA‐2	302	313	149	140	158.6	[61.0; 597.3]	743.3	[350.8; 122239.4]	207.6	[84.0; 861.4]	699.2	[327.3; ∞]	768.3	[257.4; ∞]	626.9	[304.4; ∞]
GB‐1	25	51	15	15	94.8	[19.8; ∞]	23.1	[17.1; 33.7]	241.1	[22.3; ∞]	32.2	[22.1; 55.1]	272.8	[21.2; ∞]	20.0	[14.8; 30.7]
GB‐2	323	690	192	150	534.4	[144.5; ∞]	278	[202.2; 419.5]	409.8	[114.1; ∞]	255.8	[187.2; 380.5]	247.1	[98.6; ∞]	277.9	[199.2; 459.3]
GB‐3	0	111	0	60	NA	NA	NA	NA	NA	NA	NA	NA	NA	NA	NA	NA

After removing admixed individuals, metapopulation *N*_e_‐Bayesian clustering estimates remained almost similar, except for metapopulations GB‐2 and GB‐3 that exhibited a strong increase in *N*_e_ estimations for sibship and LD estimates, respectively (Table S6).

## 4 Discussion

### 4.1 Genetic Cohesion Between Sampling Years

Life‐history traits, such as reproductive systems or generational development times, are intimately linked to estimates of effective population size (Caballero 1994; Luikart et al. 2010; Wang 1997; Waples 2024; Waples et al. 2013). For example, the presence of overlapping generations in polygamous species, where individuals reproduce for many years, reduces the effective population size (Beletsky and Orians 1989; Chen et al. 2019). In contrast, the occurrence of seed banks in plant populations greatly reduces the loss of heterozygosity over time and thus increases effective size (Nunney 2002). These conclusions are also applicable to other organisms with similar life‐history traits such as variable diapausing time or variable maturation time. Understanding the life‐history traits of the studied species is therefore a necessary prerequisite for estimating and interpreting effective population sizes (Waples 2005; Waples et al. 2013; Waples and Yokota 2007). The development time of a generation is important because if individuals do not maintain a variation in their rate of development and if no individuals are moving from one cohort to another to maintain a certain degree of genetic cohesion, each temporal cohort will lead in the long term to independent demographic evolutionary trajectories with different numbers of breeders and distinct effective population sizes for each cohort (Bouaouina et al. 2023; Gradish et al. 2019; Taylor and Friesen 2017).

The southern damselfly voltinism varies along a latitudinal gradient (Mahdjoub et al. 2014, 2015; Purse and Thompson 2002). In addition, the development time of individuals can be decreased by 1 year by environmental factors such as industrial cooling waters increasing the water temperature (Thelen 1992). Our results showed a lack of significant levels of genetic differentiation for each population between two sampling years. Moreover, the levels of genetic differentiation measured between spatial populations remained stable over time, and the genetic structure observed in the region was mainly explained by differences between populations and not by differences between sampling years. Our results, although derived at a lower latitude and in a more central area of the species' range, were therefore consistent with previous observations conducted in the United Kingdom, towards the north of the species' range (Watts and Thompson 2012). We therefore reiterate the findings of Watts and Thompson (2012), indicating low levels of genetic divergence between sympatric cohorts as a result of developmental plasticity where a fraction of individuals complete their development outside of a 2‐year period, thus mixing alternate‐year cohorts.

### 4.2 Effects of Gene Flow on Estimation of Effective Population Size (*N*_e_)

Effective population size is an essential concept in population genetics and conservation biology because it summarises the history of the population regarding genetic drift and inbreeding, and it helps to assess and to monitor the vulnerability of populations (Allendorf et al. 2022; Frankham, Briscoe, and Ballou 2013; Wang, Santiago, and Caballero 2016; Waples 2005). Well‐defined populations are natural focal units in evolutionary biology and for species conservation (Frankham et al. 2017). Therefore, the identification of their boundaries is of particular concern and has far‐reaching consequences in terms of management, particularly because identifying the proper scale for estimating *N*_e_ is an essential prerequisite to set conservation guidelines (Palstra and Ruzzante 2008; Ryman, Laikre, and Hössjer 2019; Waples 2024; Waples and England 2011). Yet, the understanding of patterns of genetic connectivity among different groups of individuals, as well as the precise quantification of their *N*_*e*_, requires an appropriate definition of what is a population for predicting and preventing the decrease of genetic diversity through random drift effects in wild populations (Baalsrud et al. 2014; Gargiulo et al. 2023). This proper definition has a flexible nature in terms of concept, depending on whether an ecological, evolutionary, statistical or variational paradigm is considered (Waples and Gaggiotti 2006). Altogether, all definitions imply a cohesive process that merges individuals within a given population. In our study, we will retain the most consensual definition from an evolutionary perspective, that is, the one involving a set of individuals living in sufficiently close geographical proximity so that successful mating events among those individuals are expected (Waples and Gaggiotti 2006).

However, it is worth noting that in most real‐world populations, the magnitude of departures from random mating following geographical isolation occurs along a continuum of population genetic differentiation, which makes it difficult to define at which point the different subunits can be properly classified as distinct populations, especially in the context of real metapopulations that will typically be more complex and subjected to local extinction events (Gilbert and Whitlock 2015; Kurland et al. 2023; Neel et al. 2013; Waples and Gaggiotti 2006). This may be especially true in our case study where relatively low genetic differentiation among populations occurred (mean *F*_ST_ of 0.022 and 0.019 for microsatellite and SNP markers respectively) and where isolation by distance takes place (see Lévêque, Duputié, et al. 2024). Thus, estimating *N*_e_ in continuously distributed species, like the southern damselfly in the neighbourhood of the Strasbourg city, with subtle genetic boundaries occurring, may present strong challenges for any methods aiming at estimating *N*_e_ (Gilbert and Whitlock 2015; Santos‐del‐Blanco et al. 2022; Waples 2024). Our results illustrated the difficulty of thoroughly estimating local effective population sizes in southern damselfly populations and highlighted the need to define proper scale upon which metapopulation *N*_e_ can be estimated.

In this context of continuously distributed populations, it should be emphasised that *N*_e_ was originally defined for a single and isolated population of constant size (Wright 1931, 1943; reviewed in Ryman, Laikre, and Hössjer 2019; Waples 2024). Nonetheless, underlying models for both one‐ or two‐sample methods for estimating *N*_e_ typically involve a number of simplifying assumptions, such as molecular neutrality of markers used, discrete generations, variance in mating success among individuals, unlinked loci or immigration‐closed populations. All of these assumptions are not satisfied in real‐world populations and have confounding effects on *N*_e_ estimation. Among those factors, the effect of genetically structured populations undergoing migration with no clear genetic discontinuities, and possibly extinctions, received ample attention both from an empirical (e.g., Baalsrud et al. 2014; Gargiulo et al. 2023) and theoretical point of view (e.g., Gilbert and Whitlock 2015; Kurland et al. 2023; Neel et al. 2013; Palstra and Ruzzante 2008; Ryman, Laikre, and Hössjer 2019; Waples and Do 2010; Waples and England 2011). Indeed, *N*_e_ is intimately related to the rate at which random genetic drift effects occur, but migration can also modify allele frequencies over time and change the amounts of LD (Gilbert and Whitlock 2015; Neel et al. 2013; Ryman, Laikre, and Hössjer 2019; Waples 2024). Migration thus complicates the quantification of drift and violations of the standard assumptions that focal studied populations are closed to immigration events may lead to biases in *N*_e_ estimates, especially when the delineation of population boundaries are difficult to depict, when isolation by distance is occurring or when the sampling size does not match the mean geographical distance over which random mating occurs (Gilbert and Whitlock 2015; Neel et al. 2013).

Gene flow can affect single‐sample and temporal estimates of *N*_e_ in contrasting ways, with the direction of bias depending both on the relative magnitude and continuity of gene flow, and also on whether immigrant individuals are genetically divergent or not (Gilbert and Whitlock 2015; Neel et al. 2013; Palstra and Ruzzante 2008; Waples 2024; Waples and England 2011). On one hand, signature of larger amounts of drift due to genetically divergent migrants will create temporal variance in allele frequencies and mixture LD due to population mixture or admixture that create a Wahlund effect. This is expected to depress estimates of local *N*_e_. Given the low levels of genetic differentiation observed among southern damselfly populations over the studied area, we could expect that such a downwardly bias in *N*_e_ estimations was not likely to arise.

On the other hand, under low levels of genetic differentiation among populations, migration is expected to provide a larger number of parents than actually exist within the focal local populations (Gilbert and Whitlock 2015; Waples and England 2011). Thereby, this may yield to a decrease in genetic drift effects within local samples that leads to an upward bias in *N*_e_ estimations, because migration expands the total number of parents. This upward bias in local *N*_e_ estimations was likely to occur in southern damselfly populations because of the low levels of genetic differentiation observed among populations, with pairwise *F*_ST_ ranging either from −0.013 to 0.114 and from −0.05 to 0.57 using microsatellite loci and SNP markers, respectively. By considering each local sampled population as independent units not connected by gene flow events, we found a large proportion of infinite estimates of local *N*_e_, whatever the single and temporal method used, which cannot be considered biologically meaningful to set conservation guidelines. Besides, this makes the estimation of effective size imprecise because it remains difficult to disentangle whether an infinite estimate of *N*_e_ actually depicts a large *N*_e_ or a lower *N*_e_ that could not be correctly estimated owing to insufficient population sampling size or low number of molecular markers used (Clarke et al. 2024; Waples 2024). In such a case, we cannot distinguish whether the local populations were truly large enough not to experience detectable genetic drift effects or if the single or temporal methods we used were unable to determine an accurate *N*_e_ (Clarke et al. 2024; Gilbert and Whitlock 2015; Waples 2024). It should be noted that, beyond the effect of migration, our results pointed out that *N*_e_ estimates based on individual relatedness (see Wang 2009) with microsatellite markers did not present the problem of infinite effective size as a signature of upwardly biased *N*_e_ estimates owing to non‐trivial migration events. Instead, this method showed somewhat low and constant estimates of local effective population size (ranging from 19 to 44). Lower estimates on *N*_e_ with sibship estimates were already observed using microsatellite data (e.g., Woltmann et al. 2019) and the discrepancies observed in our results could be explained by additional violations of the assumptions associated with this estimator, such as random sampling of individuals, or, alternatively, could be explained by the lack of resolutive information from the microsatellite markers we used (Wang 2004, 2009). Nonetheless, using the sibship estimator on population grouping based on Bayesian clustering or based on non‐significant pairwise *F*_ST_ led to a substantial increase in metapopulation *N*_e_ estimates, especially using the SNP dataset for which we depicted large metapopulation *N*_e_, ranging from 150 to infinite estimates, depending on the population grouping considered. Along with departures from the closed‐population expectation, this probably reflects a strong sensitivity of this estimator to both sampling size and marker resolution.

Nonetheless, Waples and England (2011) showed that the LD method is relatively robust to equilibrium migration, with effects of additional parents under migration being small unless the migration rate is large. Indeed, substantial upward biases in local *N*_e_ are not expected and LD estimates of *N*_e_ accurately reflecting local *N*_e_ unless the migration rate (*m*) is higher than 5%–10% (Waples 2024; Waples and England 2011). Otherwise, *N*_e_ will converge toward the global metapopulation *N*_e_ (see also Ryman, Laikre, and Hössjer 2019, for the temporal approach). In this respect, and as suggested by Waples and England (2011), we accounted for the effects of spatially structured populations thought to be connected by non‐negligible migration by considering sets of non‐genetically differentiated populations, and by considering the occurrence of genetic discontinuities and population boundaries to reanalyse *N*_e_ at the metapopulation level. Keeping in mind that pooling individuals from diverse genetic neighbourhoods should be interpreted with caution (see Kardos and Waples 2024; Neel et al. 2013), this meant that when the migration rate is substantial (> 10%), *N*_e_ estimates of local populations mirrored metapopulation *N*_e_ rather than local *N*_e_ (Waples 2024). Results from metapopulation *N*_e_–*F*_ST_ and metapopulation *N*_e_‐Bayesian clustering were congruent among markers and estimators, suggesting that *N*_e_ estimates at the scale of global metapopulations were not confounded by migration effects as it was the case for local populations (see however the local southern damselfly population named S21 that displayed a peculiar genetic structure). Besides, most metapopulations *N*_e_ were higher than 500, which could reflect relatively safe populations from a short‐term conservation genetics point of view and corroborates the general view that population subdivision increases the overall effective size of the metapopulation (Wright 1943). Altogether, these metapopulation genetic estimates of *N*_e_ are in close agreement with the very large direct estimations of population sizes in the southern damselfly using mark–release–recapture field work conducted by Allen and Thompson (2014) in southern England. Indeed, Allen and Thompson (2014) found estimated population sizes that were much larger than previously thought, ranging from 1262 to more than 60,000 individuals. This suggests that where habitats are effectively maintained and managed to form a continuous network of suitable freshwater streams, populations of threatened Odonates can be very large, with efficient recolonisation of empty locations within large clusters of favourable habitats through frequent dispersal events (Allen and Thompson 2014; Purse et al. 2003; Watts, Saccheri, et al. 2007). As immigration has a positive effect on the *N*_e_/*N*_c_ ratio, facilitating gene flow in fragmented habitats is a common measure to reduce the loss of genetic diversity, but this conservation action may not be needed in the focal region.

To temperate this positive biological conservation point, it should be noted, however, that sampling size is also known to affect temporal *N*_e_ estimates because allele frequency shifts depict not only random genetic drift but also the effect of sampling process (Neel et al. 2013; Palstra and Ruzzante 2008; Waples 1989). This effect is expected to be particularly crucial for species characterised by large census size, for which a large number of individuals should be sampled to override the effects of sampling variance and to obtain reliable estimates of *N*_e_ (Palstra and Ruzzante 2008). As in such a case the genetic drift is weaker and more difficult to detect, this may explain why when using microsatellite loci, infinite estimates of *N*_e_ were found for local *N*_e_ and metapopulation *N*_e_–*F*_ST_, also presumably because of low marker resolution. Alternatively, it may also imply the occurrence of more migrant individuals because of larger sampling sizes used with microsatellite loci. Nonetheless, metapopulation *N*_e_ based on Bayesian clustering yielded consistently finite estimates of *N*_e_, with for instance large temporal and single‐sample *N*_e_ estimates for the GA‐2 grouping, and moderate temporal and single‐sample *N*_e_ estimates for the GB‐1 grouping, which in this latter case may reflect the effect of a lower number of sampled individuals. All things being equal and in terms of applied conservation and management, most of the metapopulations we defined can therefore be considered as displaying large *N*_e_ and did not deserve strong conservation measures to mitigate anthropogenic pressures, except grouping G2 based on non‐significant pairwise *F*_ST_ for which the lowest metapopulation *N*_e_ estimates were found.

### 4.3 Local Impact of Urbanisation on Southern Damselfly Effective Population Sizes

Using Bayesian clustering and based on pairwise genetic differentiation levels, the S21 population located directly north of the city of Strasbourg appeared as the only single genetic entity isolated from all other populations. This population showed very low estimated effective population sizes with local *N*_e_ values ranging from 41.5 and 52, depending on the *N*_e_ estimator. This low effective size could be the signature of a high geographical isolation along with low census size. Paradoxically, this local population also exhibited a high level of genetic diversity (Lévêque, Duputié, et al. 2024). This peculiar within‐population structure may then reflect a sink population receiving genetically divergent individuals.

Besides, apart from this local sampling site, the GB‐1 metapopulation, also located in the direct vicinity of Strasbourg city, showed low single‐sample and temporal estimates of *N*_e_. Thus, and beyond a simple sampling size effect, specific local and metapopulation located in the direct neighbourhood of Strasbourg city may face a negative urban effect depressing *N*_e_, which is in line with an increasing effect of genetic isolation for populations close to the city centre (Lévêque, Duputié, et al. 2024).

## 5 Conclusion and Perspectives

Because *N*_e_ is used to set conservation and management guidelines, it is crucial to be cautious in interpreting *N*_e_ estimates, and using different markers along with single and temporal estimators can help to define relevant conservation actions. This study confirmed that there was no genetic isolation between sampling years in southern damselflies, suggesting plasticity in the semi‐voltine trait of the species and, consequently, no independent evolutionary trajectories of temporally spaced and genetically differentiated cohorts (see also Thelen 1992; Watts and Thompson 2012). *N*_e_ is a pivotal evolutionary parameter for monitoring and predicting the standing genetic variation driving the evolutionary potential of wild populations. Although this empirical study illustrated the inherent difficulty of accurately estimating effective population sizes, even using a large set of different genomic markers expected to yield greater accuracy than a microsatellite‐based dataset, our results stressed the importance of properly defining genetic discontinuities in continuously distributed populations displaying low genetic differentiation before attempting to estimate *N*_e_ to inform conservation decisions. Continuously distributed southern damselfly populations located in the Alsace region appear to be, at the present time, safe in terms of effective population size and do not deserve strong conservation measures apart from maintaining their habitats. Nonetheless, highly urbanised areas may have a local negative impact in terms of population genetic features and *N*_e_ for populations located in the close vicinity of the city of Strasbourg, as expected under an ‘urban fragmentation model’ (Miles et al. 2019).

## Conflicts of Interest

The authors declare no conflicts of interest.

## Supporting information


Data S1.

## Data Availability

Data for this study are available on Dryad (https://doi.org/10.5061/dryad.83bk3jb34).
