# Supplementary material for: Levels and Spatial Patterns of Effective Population Sizes in the Southern Damselfly (Coenagrion mercuriale): On the Need to Carefully Interpret Single‐Point and Temporal Estimations to Set Conservation Guidelines
Source: Evol Appl. 2024 Dec 24;17(12):e70062. doi: 10.1111/eva.70062 (PMC11667679; doi:10.1111/eva.70062)
Supplement: Supplementary file 1 — Data S1. [file EVA-17-e70062-s001.docx]

Supplementary file for “**Levels and spatial patterns of effective population sizes in the southern damselfly (*Coenagrion mercuriale*): on the need to carefully interpret single-point and temporal estimations to set conservation guidelines**.”


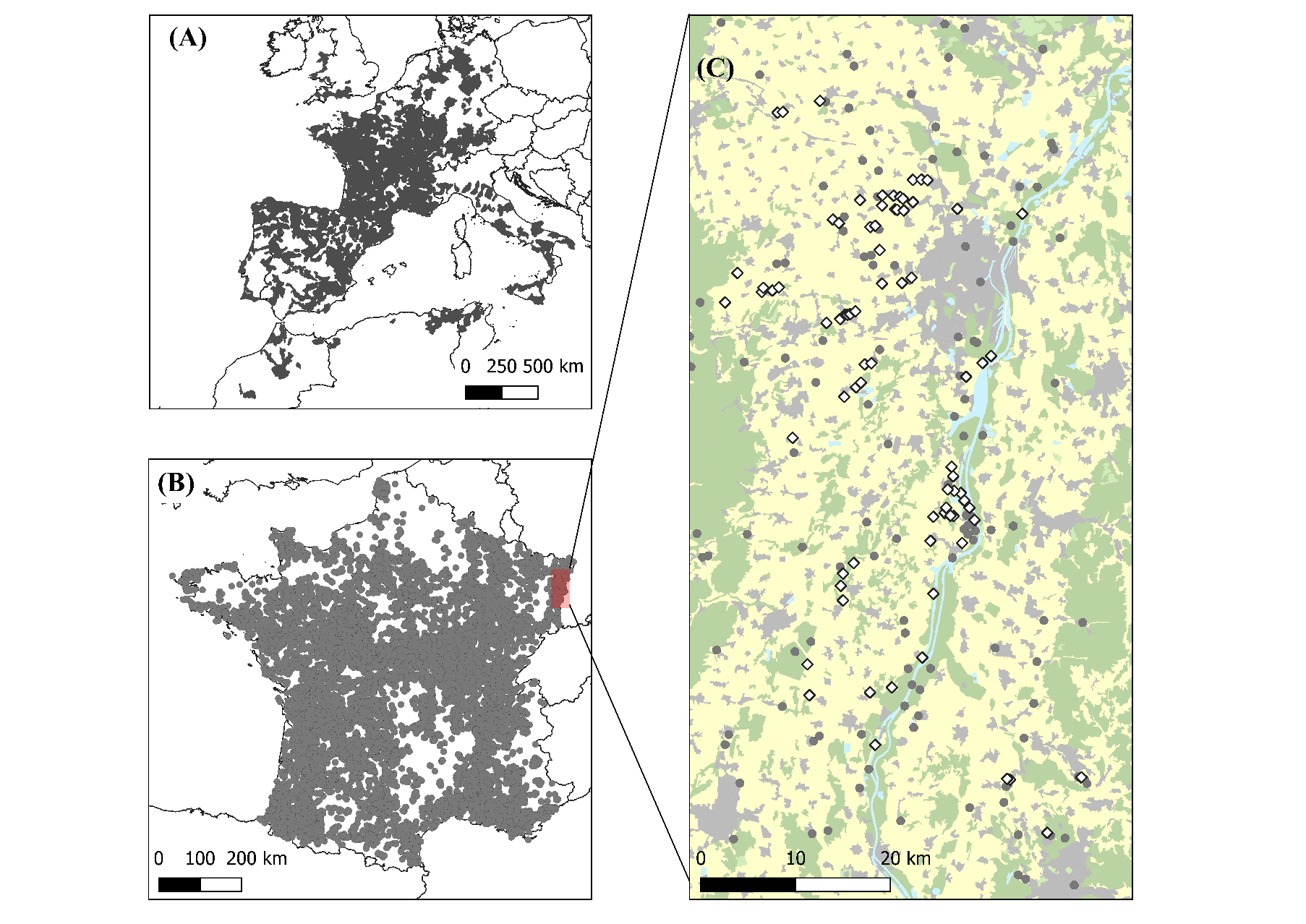


#
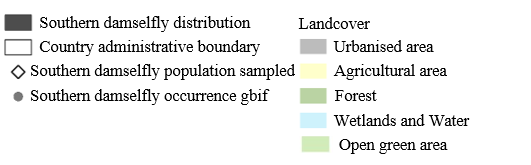


Figure S1**: (A)** Map showing the current geographical distribution (dark grey zones) of the southern damselfly (*Coenagrion mercuriale*). Black lines indicate the administrative limits of countries. Data obtained from IUCN SSC Odonata Specialist Group 2019. The IUCN Red List of Threatened Species. Version 2022-2. https://www.iucnredlist.org/ Downloaded on 28 July 2023. **(B)** Map showing the occurrences of southern damselfly recorded in France (grey dots). The study zone in panel B is highlighted in red. Data obtained from GBIF.org (5 March 2024) GBIF occurrence downloaded at https://doi.org/10.15468/dl.5z9gue. **(C)** Geographical location of southern damselfly sampling sites in north-eastern France (white diamonds). Land cover was simplified from Corine Land Cover Edition 2018. Grey dots indicate occurrences of southern damselfly recorded in France and Germany and obtained from GBIF.org (5 March 2024; https://doi.org/10.15468/dl.9h9maw).


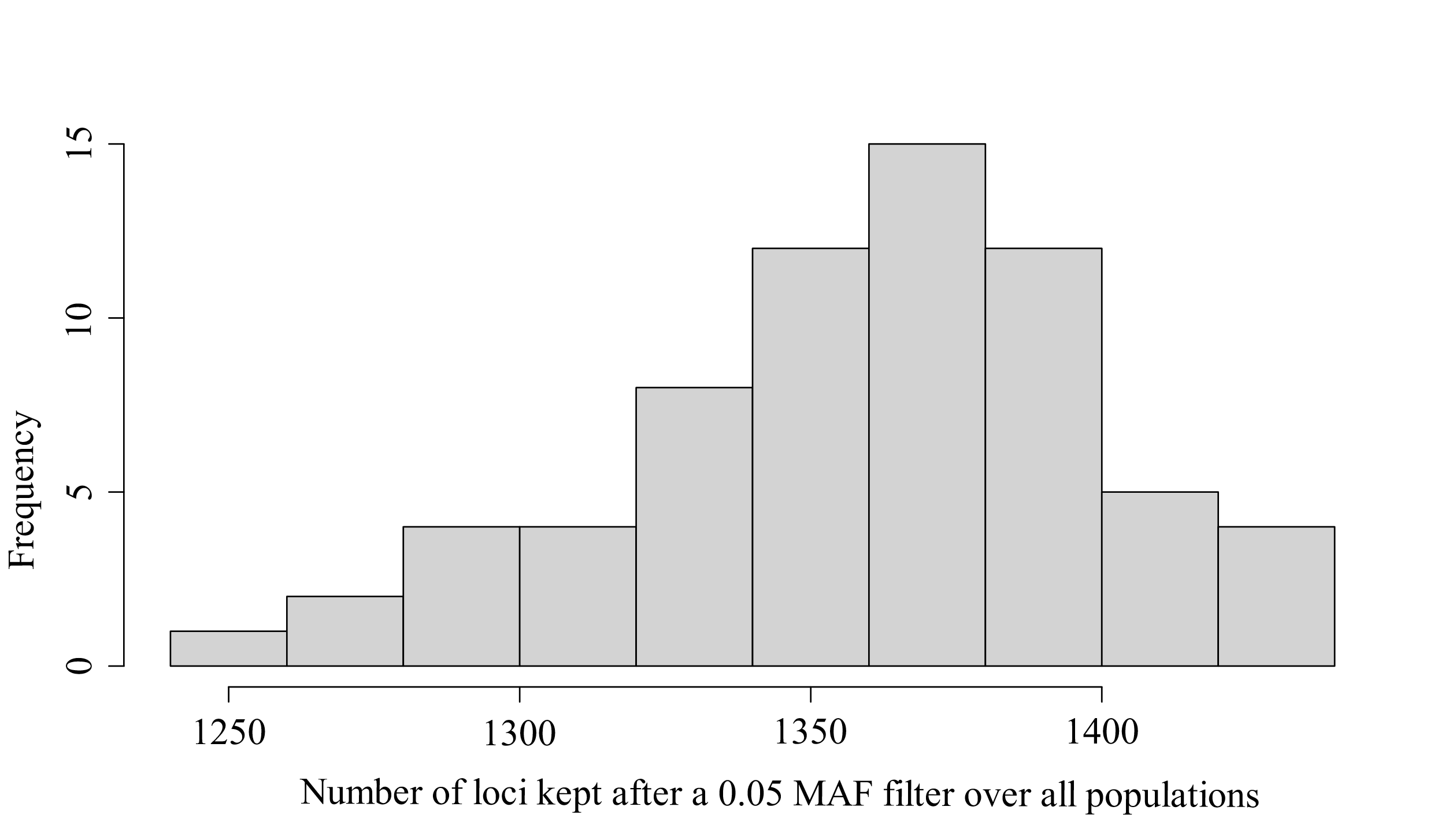


Figure S2**:** Histogram of the number of SNP loci per population used for estimations of effective population sizes (*N*_e_) after applying a 5% threshold for minor allele frequencies.


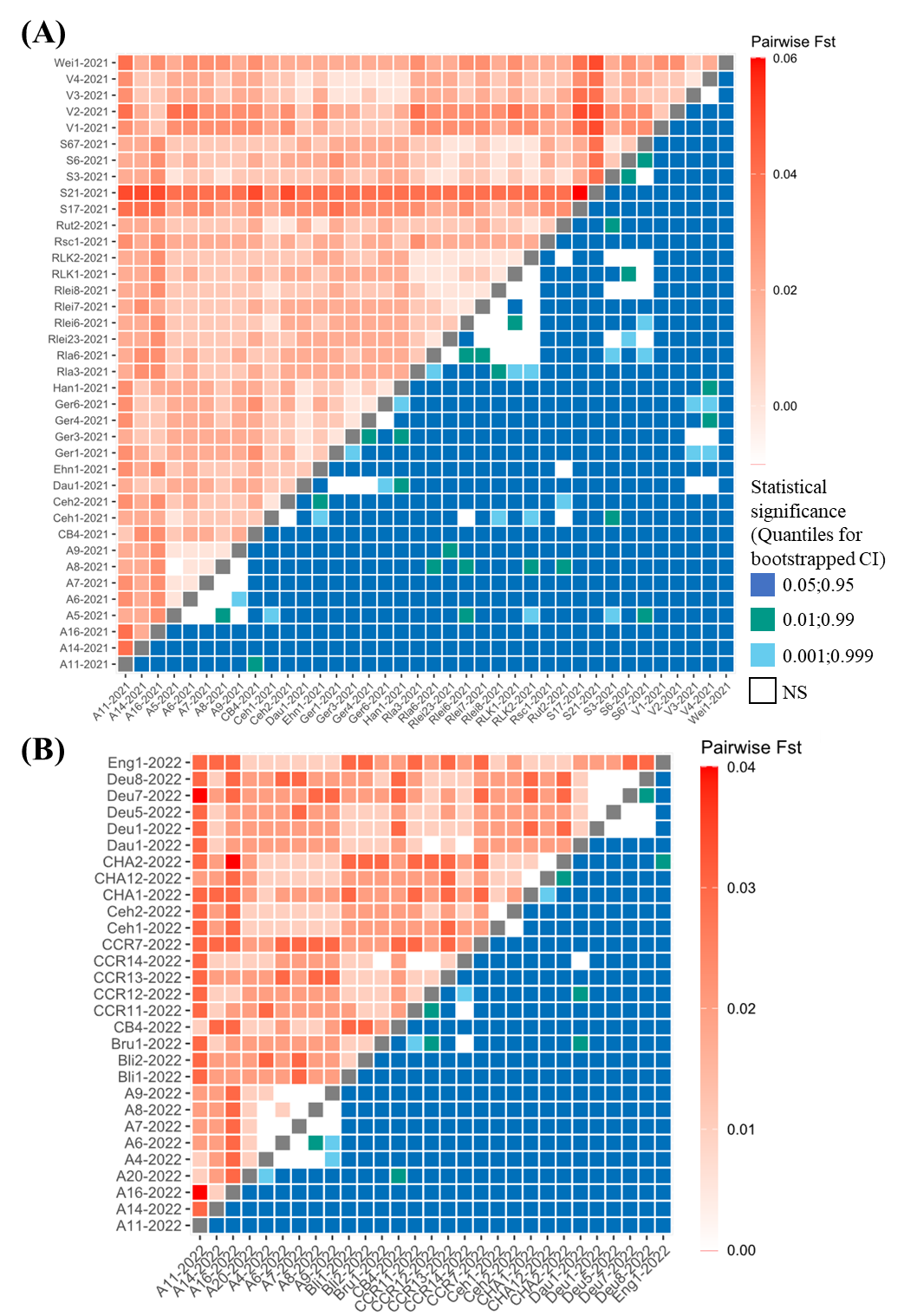
 Figure S3: Matrices of pairwise *F*_ST_ estimates (upper triangle) and their associated statistical significance assessed when confidence intervals of 1,000 bootstrap replicates did not overlap with zero (lower triangle) for populations sampled in 2021 **(A)** or in 2022 **(B)**, using the SNP dataset.


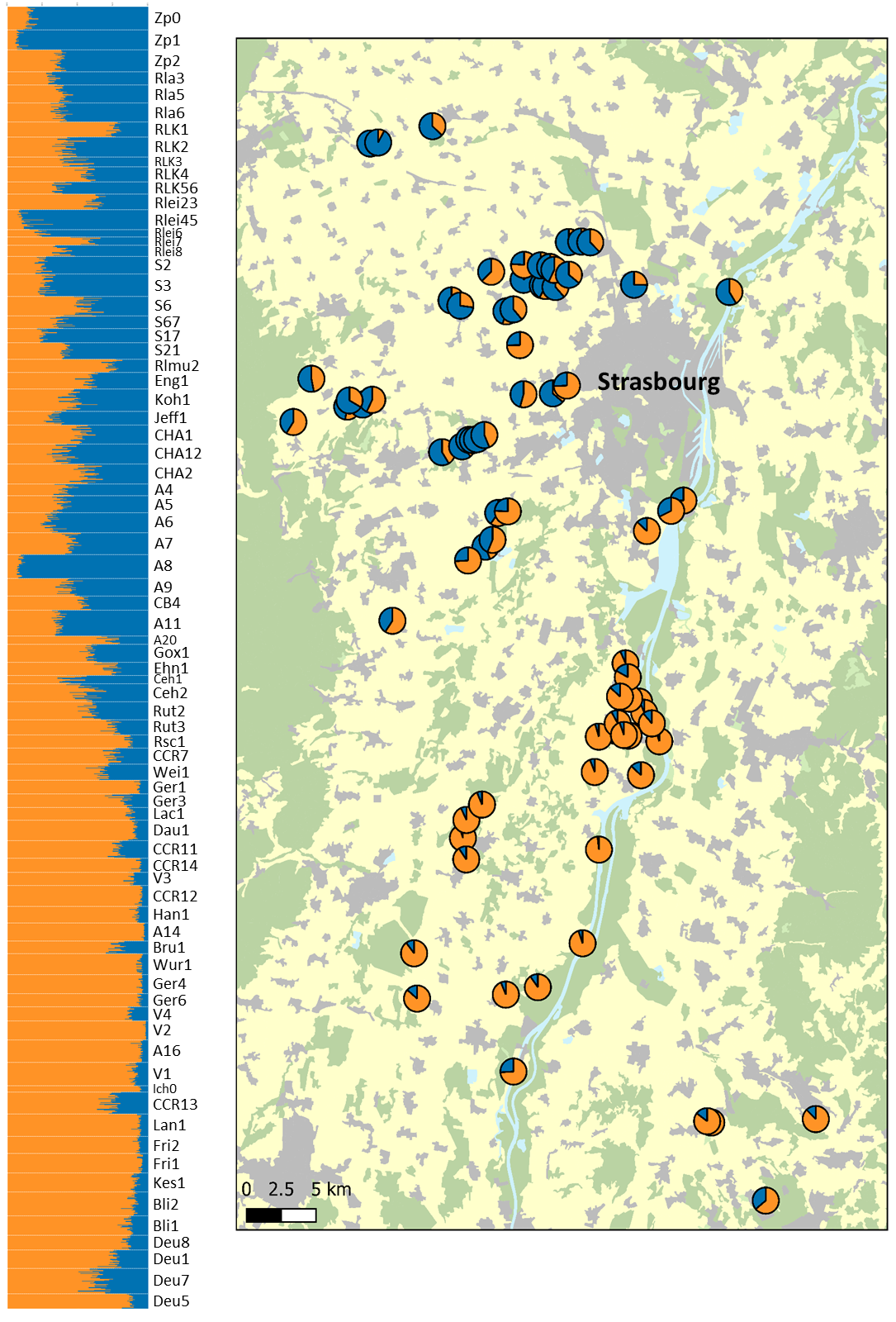
Figure S4: Bayesian clustering results obtained using Structure v.2.3.3 (Pritchard et al., 2000) applied on microsatellite data for the optimal value of K= 2. The left part illustrates bar plots showing individual membership probabilities to one of the two cluster (each cluster is represented by a different colour, and each horisontal bar represents and individuals) and right part depicts the corresponding pie charts of individual assignments for each sampling location of southern damselfly population, different colours represent different clusters assigned to populations.


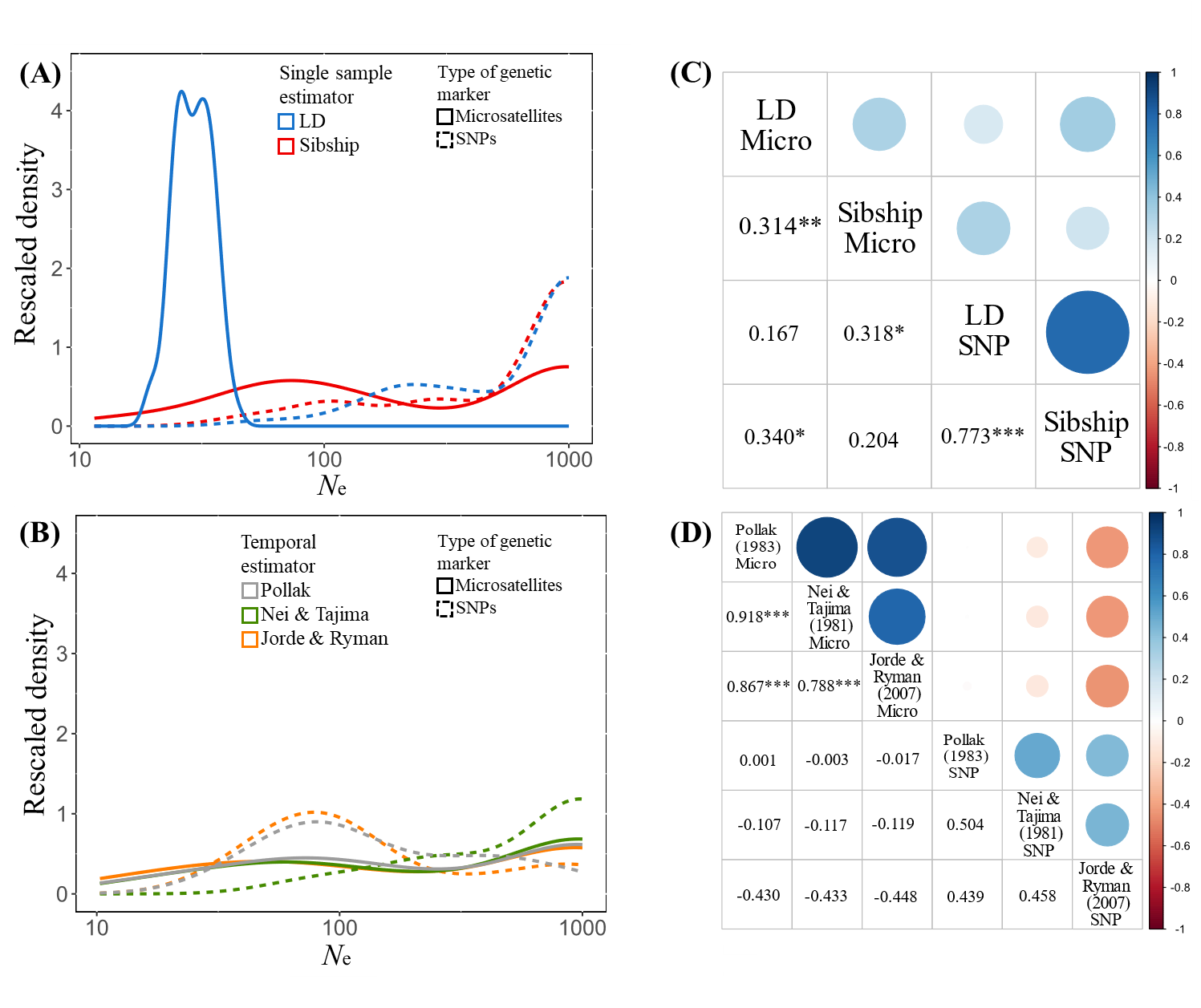


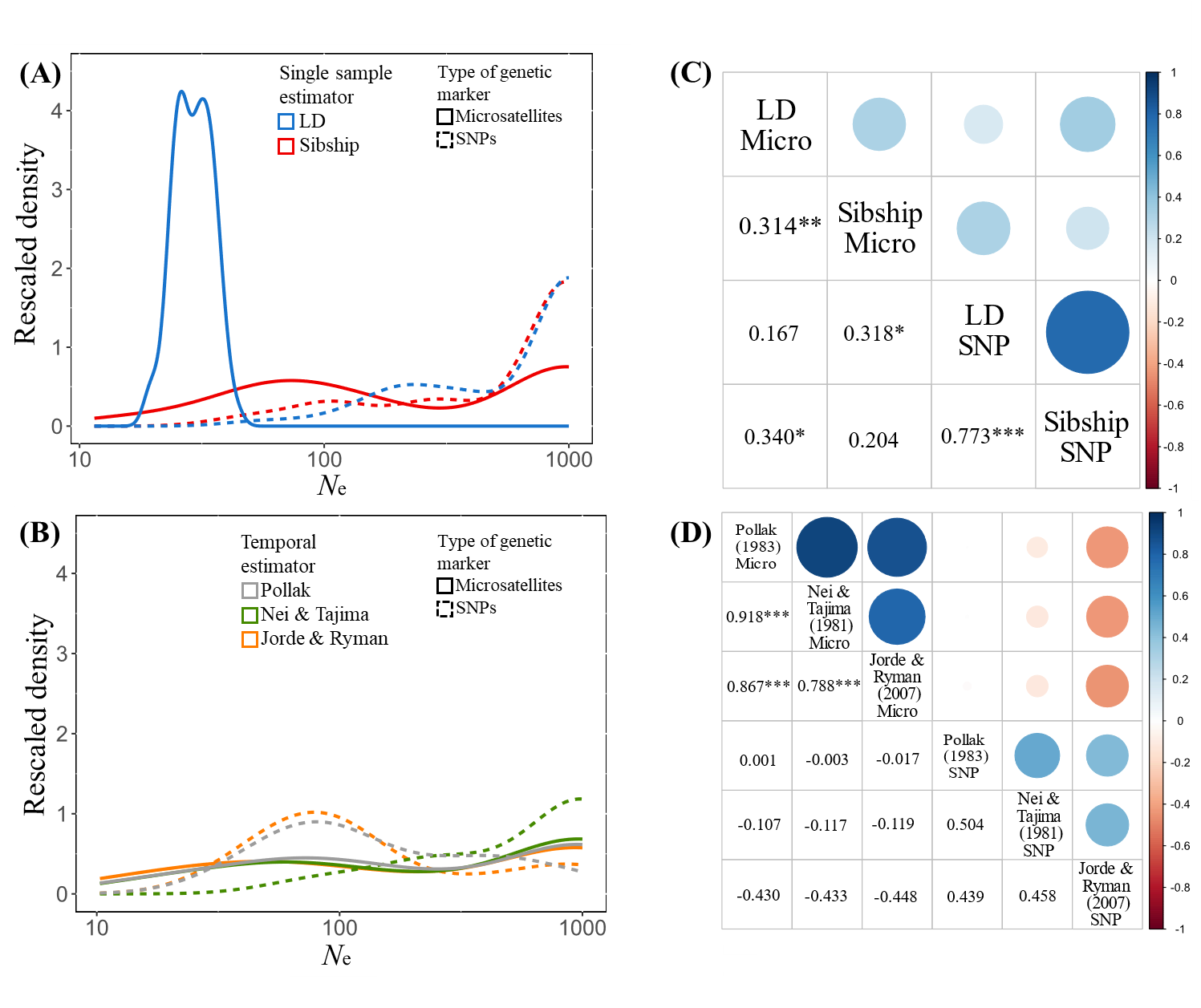


Figure S5: Density plots showing the distribution of local effective population size (*N*_e_) estimates in the southern damselfly (*Coenagrion mercuriale)*. **(A)** Single-sample methods (LD: Linkage disequilibrium; Sibship: Full-Likelihood analysis based on inferred sibship frequencies among samples) and **(B)** temporal methods using *F*_s_ (Jorde & Ryman 2007), *F*_e_ Nei & Tajima (1981), and *F*_k_ Pollak (1983) for the microsatellite (Microsatellites, solid lines) and the SNP datasets (SNPs, dotted lines). For graphical representation purposes, populations with either infinite *N*_e_ estimates or effective population sizes estimated at more than 1,000 have been set at an estimate of the effective population size of 1,000.


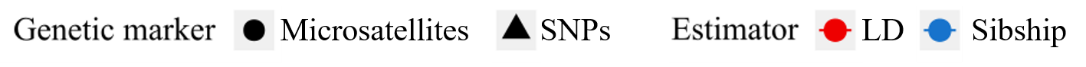


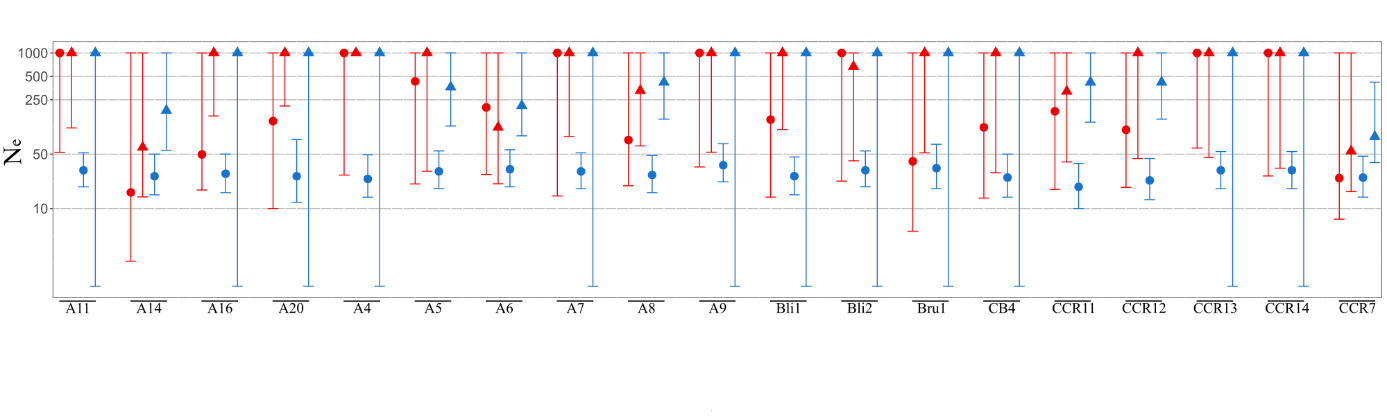

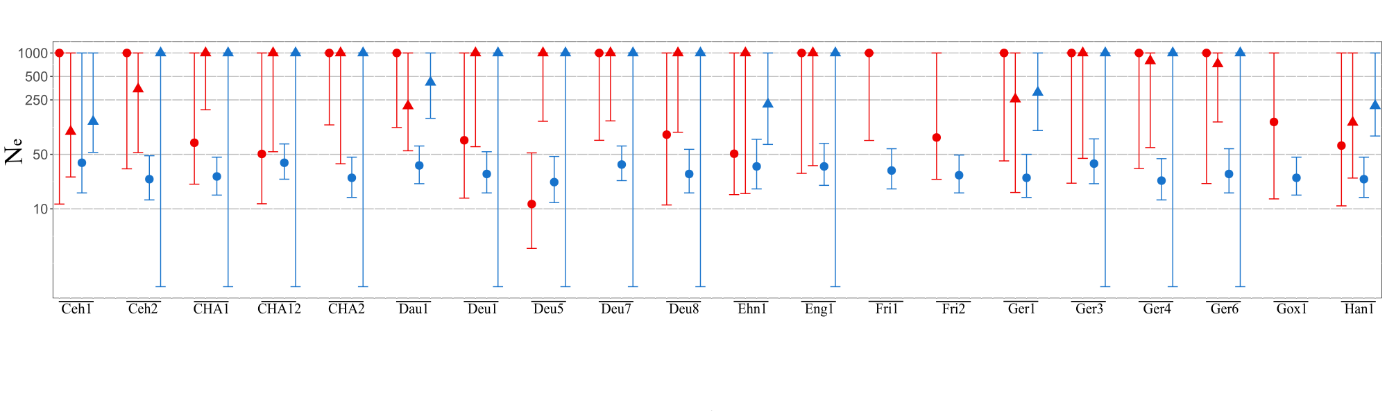


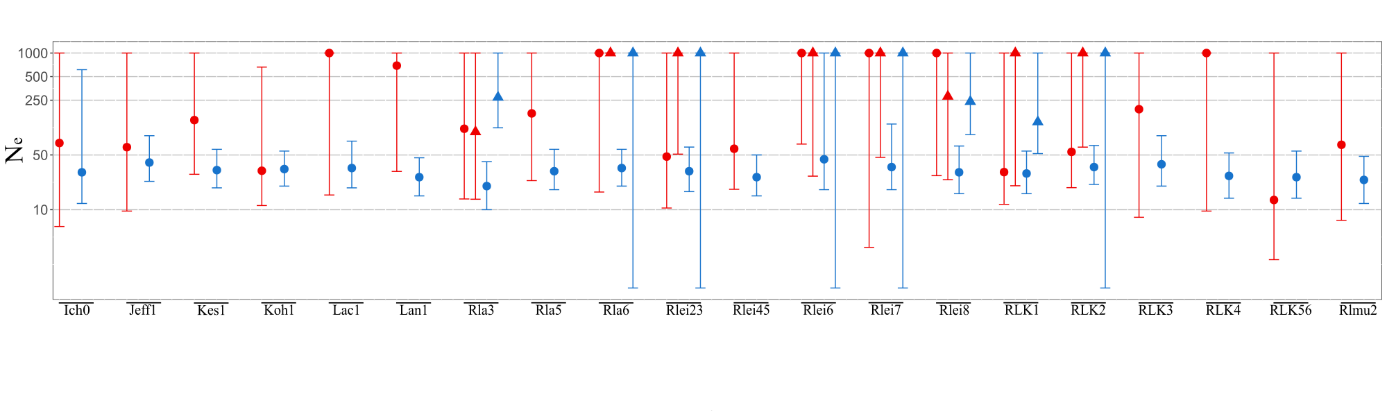

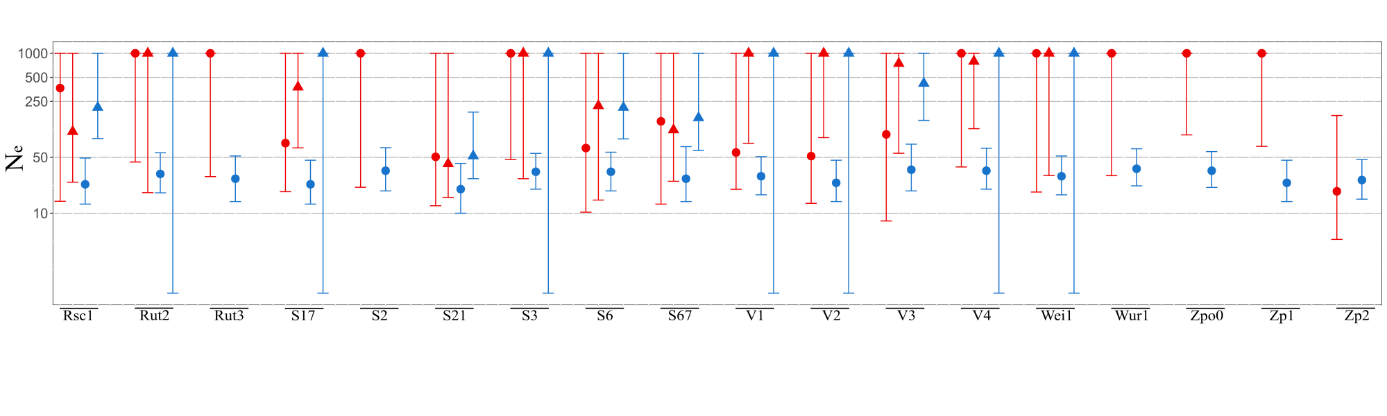


Figure S6**:** Contemporary effective population size (*N*_e_) estimates based on Linkage Disequilibrium (LD, red) and sibship assignments (Sibship, blue) generated using microsatellites (circles) for 77 southern damselfly populations and SNPs (triangles) for 56 populations. Jackknifed-based confidence intervals for LD estimation and bootstrap resampling confidence intervals for Full-Likelihood analysis are shown. Infinite estimates of *N*_e_ are represented by values of 1,000. For graphical representation purposes, populations with either infinite *N*_e_ estimates or effective population sizes estimated at more than 1,000 have been set at an estimate of the effective population size of 1,000.


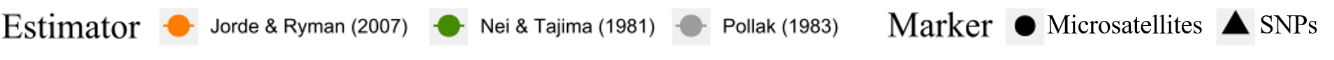

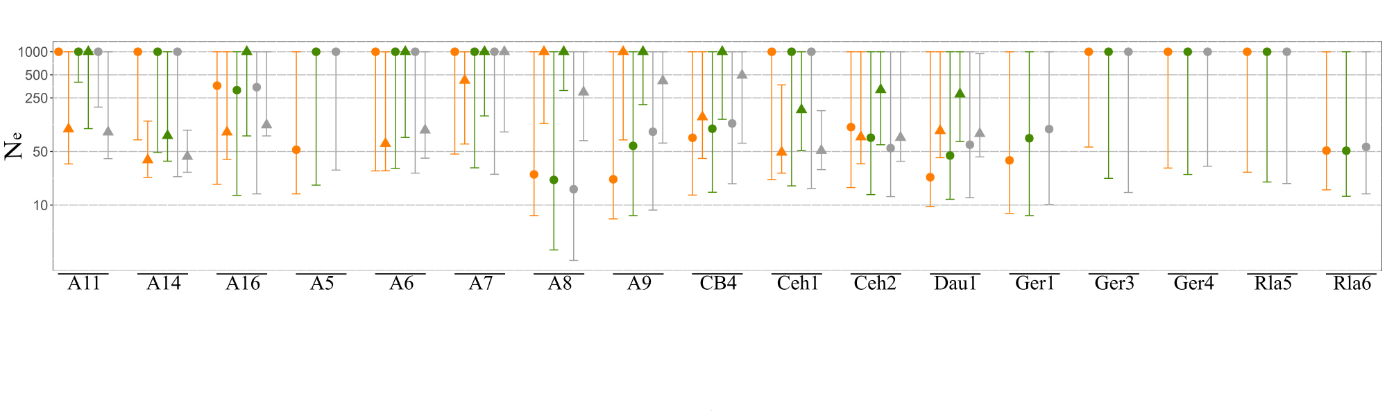

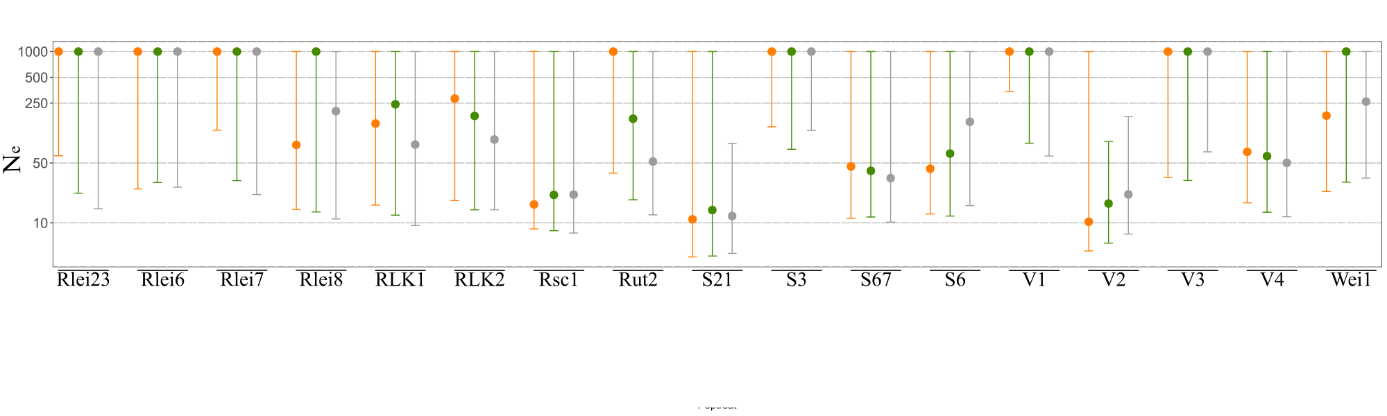


Figure S7**:** Contemporary effective population size (*N*_e_) estimates and jackknifed-based confidence intervals based on temporal estimates of Jorde & Ryman (2007, orange), of Nei & Tajima (1981, green) and of Pollak (1983, grey) generated using microsatellites (circles) for 34 southern damselfly populations and using SNPs (triangles) for 11 populations. Infinite estimates of *N*_e_ are represented by values of 1,000. For graphical representation purposes, populations with either infinite *N*_e_ estimates or effective population sizes estimated at more than 1,000 have been set at an estimate of the effective population size of 1,000.

Table S1**:** Population sampling information for southern damselfly sampled populations. Population names and geographic coordinates of the sampling sites (WGS84) are indicated, with the numbers of samples per year genotyped with microsatellite markers, and the number of samples per year genotyped with SNP markers.

|  |  |  | Microsatellites |  |  | SNPs |  |
| --- | --- | --- | --- | --- | --- | --- | --- |
|  |  |  | Sampling year and number of individuals genotyped using microsatellite loci | |  | Sampling year and number of individuals genotyped using SNP loci | |
| Population name | Longitude EPSG4326 | Latitude EPSG4326 | 2021 | 2022 |  | 2021 | 2022 |
| A4 | 7.544702 | 48.540918 | *NA* | 18 |  | *NA* | 14 |
| A5 | 7.564511 | 48.543905 | 26 | 13 |  | 15 | *NA* |
| A6 | 7.571759 | 48.547882 | 30 | 26 |  | 15 | 14 |
| A7 | 7.575255 | 48.547574 | 33 | 29 |  | 15 | 15 |
| A8 | 7.579292 | 48.548017 | 30 | 36 |  | 15 | 15 |
| A9 | 7.586779 | 48.550395 | 31 | 27 |  | 13 | 15 |
| A11 | 7.655745 | 48.574642 | 31 | 39 |  | 13 | 15 |
| A14 | 7.697191 | 48.354712 | 27 | 23 |  | 14 | 15 |
| A16 | 7.653206 | 48.218936 | 30 | 34 |  | 15 | 15 |
| A20 | 7.669929 | 48.579161 | *NA* | 13 |  | *NA* | 10 |
| Bli1 | 7.490202 | 48.189452 | *NA* | 30 |  | *NA* | 15 |
| Bli2 | 7.489811 | 48.218759 | *NA* | 36 |  | *NA* | 15 |
| Bru1 | 7.709551 | 48.351107 | *NA* | 19 |  | *NA* | 15 |
| CB4 | 7.627223 | 48.575197 | 18 | 21 |  | 14 | 15 |
| CCR11 | 7.721753 | 48.372592 | *NA* | 27 |  | *NA* | 15 |
| CCR12 | 7.739052 | 48.346225 | *NA* | 32 |  | *NA* | 15 |
| CCR13 | 7.579254 | 48.138813 | *NA* | 33 |  | *NA* | 15 |
| CCR14 | 7.726147 | 48.365158 | *NA* | 21 |  | *NA* | 15 |
| CCR7 | 7.776558 | 48.500519 | *NA* | 24 |  | *NA* | 15 |
| CHA1 | 7.454961 | 48.573777 | *NA* | 29 |  | *NA* | 15 |
| CHA12 | 7.470093 | 48.574551 | *NA* | 30 |  | *NA* | 15 |
| CHA2 | 7.479667 | 48.577296 | *NA* | 30 |  | *NA* | 15 |
| Ceh1 | 7.581641 | 48.478382 | 31 | 13 |  | 11 | 12 |
| Ceh2 | 7.588845 | 48.482536 | 25 | 27 |  | 12 | 15 |
| Dau1 | 7.712416 | 48.374919 | 26 | 31 |  | 15 | 15 |
| Deu1 | 7.767138 | 48.098375 | *NA* | 28 |  | *NA* | 15 |
| Deu5 | 7.868078 | 48.096462 | *NA* | 23 |  | *NA* | 15 |
| Deu7 | 7.815028 | 48.045885 | *NA* | 38 |  | *NA* | 15 |
| Deu8 | 7.763198 | 48.099368 | *NA* | 22 |  | *NA* | 15 |
| Ehn1 | 7.563860 | 48.470101 | 20 | *NA* |  | 11 | *NA* |
| Eng1 | 7.401690 | 48.565821 | *NA* | 25 |  | *NA* | 11 |
| Fri1 | 7.543433 | 48.291327 | *NA* | 29 |  | *NA* | *NA* |
| Fri2 | 7.547780 | 48.302849 | *NA* | 26 |  | *NA* | *NA* |
| Ger1 | 7.710864 | 48.397795 | 20 | 33 |  | 13 | *NA* |
| Ger3 | 7.712247 | 48.388783 | 21 | 12 |  | 15 | *NA* |
| Ger4 | 7.680718 | 48.351555 | 29 | 30 |  | 15 | *NA* |
| Ger6 | 7.674729 | 48.328929 | 20 | *NA* |  | 15 | *NA* |
| Gox1 | 7.487110 | 48.434042 | *NA* | 27 |  | *NA* | *NA* |
| Han1 | 7.699740 | 48.359411 | 25 | *NA* |  | 15 | *NA* |
| Ich0 | 7.576146 | 48.188722 | *NA* | 10 |  | *NA* | *NA* |
| Jeff1 | 7.457562 | 48.577673 | *NA* | 21 |  | *NA* | *NA* |
| Kes1 | 7.545415 | 48.277385 | *NA* | 29 |  | *NA* | *NA* |
| Koh1 | 7.421644 | 48.593061 | *NA* | 34 |  | *NA* | *NA* |
| Lac1 | 7.703560 | 48.376597 | *NA* | 19 |  | *NA* | *NA* |
| Lan1 | 7.563761 | 48.312189 | *NA* | 34 |  | *NA* | *NA* |
| Rla3 | 7.680047 | 48.671614 | 20 | *NA* |  | 17 | *NA* |
| Rla5 | 7.692171 | 48.671474 | 21 | 27 |  | *NA* | *NA* |
| Rla6 | 7.700949 | 48.670594 | 29 | 28 |  | 12 | *NA* |
| Rlei23 | 7.602713 | 48.655404 | 24 | 15 |  | 15 | *NA* |
| Rlei45 | 7.633774 | 48.649009 | *NA* | 30 |  | *NA* | *NA* |
| Rlei6 | 7.652179 | 48.644755 | 11 | 29 |  | 11 | *NA* |
| Rlei7 | 7.654957 | 48.644172 | 13 | 34 |  | 12 | *NA* |
| Rlei8 | 7.664481 | 48.643140 | 17 | 26 |  | 16 | *NA* |
| RLK1 | 7.635142 | 48.658617 | 23 | 18 |  | 12 | *NA* |
| RLK2 | 7.651268 | 48.657640 | 30 | 33 |  | 13 | *NA* |
| RLK3 | 7.660315 | 48.656235 | *NA* | 15 |  | *NA* | *NA* |
| RLK4 | 7.664291 | 48.654240 | *NA* | 23 |  | *NA* | *NA* |
| RLK56 | 7.678233 | 48.650551 | *NA* | 18 |  | *NA* | *NA* |
| Rlmu2 | 7.626599 | 48.607025 | *NA* | 20 |  | *NA* | *NA* |
| Rsc1 | 7.739234 | 48.482301 | 20 | 20 |  | 15 | *NA* |
| Rut2 | 7.595870 | 48.499645 | 27 | 28 |  | 15 | *NA* |
| Rut3 | 7.605300 | 48.500283 | *NA* | 23 |  | *NA* | *NA* |
| S2 | 7.562404 | 48.638451 | *NA* | 26 |  | *NA* | *NA* |
| S3 | 7.570723 | 48.634706 | 34 | 20 |  | 15 | *NA* |
| S6 | 7.615283 | 48.629329 | 31 | 23 |  | 15 | *NA* |
| S67 | 7.622262 | 48.630331 | 18 | 20 |  | 13 | *NA* |
| S17 | 7.741153 | 48.641593 | 21 | *NA* |  | 15 | *NA* |
| S21 | 7.833577 | 48.633175 | 25 | 11 |  | 15 | *NA* |
| V1 | 7.607497 | 48.192280 | 35 | 33 |  | 15 | *NA* |
| V2 | 7.674399 | 48.278712 | 29 | 30 |  | 15 | *NA* |
| V3 | 7.732873 | 48.358055 | 20 | 33 |  | 15 | *NA* |
| V4 | 7.719338 | 48.325091 | 21 | 34 |  | 15 | *NA* |
| Wei1 | 7.763958 | 48.494377 | 25 | 27 |  | 15 | *NA* |
| Wur1 | 7.705175 | 48.351641 | *NA* | 32 |  | *NA* | *NA* |
| Zp0 | 7.491934 | 48.742628 | *NA* | 35 |  | *NA* | *NA* |
| Zp1 | 7.499699 | 48.742984 | *NA* | 30 |  | *NA* | *NA* |
| Zp2 | 7.553629 | 48.751473 | *NA* | 33 |  | *NA* | *NA* |

Table S2**:** Pairwise estimates of genetic differentiation (*F*_ST_) using microsatellites genetic markers, their *P*-values and associated statistical significance after Bonferroni correction for multiple testing among southern damselfly (*Coenagrion mercuriale*) populations from alternate-year cohorts (2021, 2022) in the studied area.

| Site | Pairwise *F*_ST_ | *P*-values | Indicative adjusted nominal level (5%) for multiple comparisons is : 0.000023 |
| --- | --- | --- | --- |
| A5 | 0.0036 | 0.71837 | NS |
| A6 | -0.0028 | 0.34688 | NS |
| A7 | -0.0005 | 0.58291 | NS |
| A8 | 0.0091 | 0.00231 | NS |
| A9 | 0.0103 | 0.46655 | NS |
| A11 | -0.0045 | 0.90697 | NS |
| A14 | -0.0095 | 0.73657 | NS |
| A16 | 0.0012 | 0.19266 | NS |
| CB4 | 0.0040 | 0.32347 | NS |
| Ceh1 | -0.0046 | 0.98247 | NS |
| Dau1 | 0.0112 | 0.14385 | NS |
| Ger1 | 0.0074 | 0.23336 | NS |
| Ger3 | -0.0083 | 0.41807 | NS |
| Ger4 | 0.0009 | 0.36354 | NS |
| Rla5 | -0.0001 | 0.30354 | NS |
| Rla6 | 0.0051 | 0.19811 | NS |
| Rlei23 | -0.0036 | 0.75287 | NS |
| Rlei6 | -0.0022 | 0.60478 | NS |
| Rlei7 | -0.0083 | 0.65524 | NS |
| Rlei8 | 0.0015 | 0.58368 | NS |
| RLK1 | 0.0017 | 0.20191 | NS |
| RLK2 | 0.0008 | 0.52163 | NS |
| Rsc1 | 0.0158 | 0.07235 | NS |
| Rut2 | -0.0010 | 0.04858 | NS |
| S3 | -0.0040 | 0.78163 | NS |
| S6 | 0.0074 | 0.13883 | NS |
| S67 | 0.0043 | 0.24408 | NS |
| S21 | 0.0207 | 0.04317 | NS |
| V1 | -0.0036 | 0.50312 | NS |
| V2 | 0.0238 | 0.05198 | NS |
| V3 | -0.0045 | 0.78632 | NS |
| V4 | 0.0040 | 0.36545 | NS |
| Wei1 | 0.0016 | 0.54860 | NS |

Table S3**:** Names of local populations grouped for estimating Metapopulation *N*_e__*F*_ST_ and Metapopulation *N*_e_-Bayesian clustering.

| Metapopulations defined with non-significant *F*_ST_ | Local populations grouped for estimating Metapopulation Ne*_F*_ST_ |
| --- | --- |
| G1 | RLK1, RLK2, Rlei23, Rlei6, Rlei7, Rlei8 S3, S6, S67 |
| G2 | A4, A5, A6, A7, A8, A9 |
| G3 | Ceh1, Ceh2 |
| G4 | Ger4, Ger6 |
| G5 | CCR12, V3, V4 |
| G6 | Deu1, Deu5, Deu7, Deu8 |

| Metapopulations defined with bayesian clustering | Local populations grouped for estimating Metapopulation Ne-Bayesian clustering |
| --- | --- |
| GA-1 | Eng1, Koh1, CHA1, CHA12, CHA2, Jeff1, Rla3, Rla5, Rla6, RLK1, RLK2, RLK3, RLK4, RLK56, Rlei23, Rlei45, Rlei6, Rlei7, Rlei8, S2, S3, S6, S67, S17, Rlmu2 |
| GA-2 | A11, A20, CB4, A4, A5, A6, A7, A8, A9, Ehn1, Ceh1, Ceh2, Rut2, Rut3 |
| GB-1 | CCR7, Wei1 |
| GB-2 | Rsc1, Ger1, Ger3, Ger4, Ger6, Lac1, Dau1, Han1, A14, Bru1, Wur1, V4, CCR11, CCR14, V3, CCR12, Lan1, Fri1, Fri2, Kes1, Bli1, Bli2, Ich0, V2, A16, V1, CCR13 |
| GB-3 | Deu1, Deu5, Deu7, Deu8 |

Table S4**:** *N_e_* estimates from single-sample estimators, using the Linkage Disequilibrium method (Hill, 1981; Waples, 2006; Waples and Do, 2010; Microsatellites and SNPs) and sibship assignments (Wang., 2009; Microsatellites and SNPs).

|  | Microsatellites | | | | |  | SNP | | | | |
| --- | --- | --- | --- | --- | --- | --- | --- | --- | --- | --- | --- |
|  |  | LD method | | Sibship assignment method | |  |  | LD method | | Sibship assignment method | |
| Pop | Sample Size | Estimated *Ne* | 95% CI | Estimated *Ne* | 95% CI |  | Sample Size | Estimated *Ne* | 95% CI | Estimated *Ne* | 95% CI |
| A4-2022 | 18 | ∞ | [26.9 ; ∞] | 24 | [14 ; 49] |  | 14 | ∞ | [∞ ; ∞] | ∞ | [1, ∞] |
| A5-2021 | 26 | 431.4 | [20.7 ; ∞] | 30 | [18 ; 55] |  | 15 | ∞ | [30.1 ; ∞] | 364 | [115, ∞] |
| A6-2021 | 30 | 199.5 | [27.4 ; ∞] | 32 | [19 ; 57] |  | 15 | 110.6 | [20.8 ; ∞] | 210 | [86, ∞] |
| A7-2021 | 33 | ∞ | [14.5 ; ∞] | 30 | [18 ; 52] |  | 15 | 1033.2 | [84.3 ; ∞] | ∞ | [1, ∞] |
| A8-2022 | 36 | 75.9 | [19.6 ; ∞] | 27 | [16 ; 48] |  | 15 | 327.9 | [63.8 ; ∞] | 420 | [141, ∞] |
| A9-2022 | 27 | ∞ | [34.2 ; ∞] | 36 | [22 ; 68] |  | 15 | 1781.6 | [53 ; ∞] | ∞ | [1, ∞] |
| A11-2022 | 39 | ∞ | [52.8 ; ∞] | 31 | [19 ; 52] |  | 15 | ∞ | [108.6 ; ∞] | ∞ | [1, ∞] |
| A14-2021 | 27 | 16.1 | [2.1 ; ∞] | 26 | [15 ; 50] |  | 15 | 61.3 | [14.1 ; ∞] | 182 | [56, ∞] |
| A16-2022 | 34 | 49.5 | [17.3 ; ∞] | 28 | [16 ; 50] |  | 15 | ∞ | [154.9 ; ∞] | ∞ | [1, ∞] |
| A20-2022 | 13 | 133 | [10 ; ∞] | 26 | [12 ; 77] |  | 10 | ∞ | [208.1 ; ∞] | ∞ | [1, ∞] |
| Bli1-2022 | 30 | 138.9 | [14 ; ∞] | 26 | [15 ; 46] |  | 15 | ∞ | [103.7 ; ∞] | ∞ | [1, ∞] |
| Bli2-2022 | 36 | ∞ | [22.5 ; ∞] | 31 | [19 ; 55] |  | 15 | 666.7 | [41 ; ∞] | ∞ | [1, ∞] |
| Bru1-2022 | 19 | 40.4 | [5.1 ; ∞] | 33 | [18 ; 67] |  | 15 | ∞ | [52 ; ∞] | ∞ | [1, ∞] |
| CB4-2022 | 21 | 110.6 | [13.6 ; ∞] | 25 | [14 ; 50] |  | 15 | ∞ | [28.8 ; ∞] | ∞ | [1, ∞] |
| CCR11-2022 | 27 | 177.8 | [17.6 ; ∞] | 19 | [10 ; 38] |  | 15 | 320.9 | [39.7 ; ∞] | 420 | [129, ∞] |
| CCR12-2022 | 32 | 102.6 | [18.7 ; ∞] | 23 | [13 ; 44] |  | 15 | ∞ | [44 ; ∞] | 420 | [141, ∞] |
| CCR13-2022 | 33 | ∞ | [59.9 ; ∞] | 31 | [18 ; 54] |  | 15 | ∞ | [45.5 ; ∞] | ∞ | [1, ∞] |
| CCR14-2022 | 21 | ∞ | [26.2 ; ∞] | 31 | [18 ; 54] |  | 15 | ∞ | [33 ; ∞] | ∞ | [1, ∞] |
| CCR7-2022 | 24 | 24.6 | [7.3 ; ∞] | 25 | [14 ; 47] |  | 15 | 54.7 | [16.5 ; ∞] | 84 | [39, 422] |
| CHA1-2022 | 29 | 70.4 | [20.7 ; ∞] | 26 | [15 ; 46] |  | 15 | 1903.4 | [186.6 ; ∞] | ∞ | [1, ∞] |
| CHA12-2022 | 30 | 50.6 | [11.6 ; ∞] | 39 | [24 ; 68] |  | 15 | 3796.4 | [54 ; ∞] | ∞ | [1, ∞] |
| CHA2-2022 | 30 | ∞ | [119.4 ; ∞] | 25 | [14 ; 46] |  | 15 | 4475.5 | [37.8 ; ∞] | ∞ | [1, ∞] |
| Ceh1-2022 | 13 | 2235.9 | [11.5 ; 1030.7] | 39 | [16 ; 16587] |  | 12 | 98.1 | [25.6 ; ∞] | 132 | [53, ∞] |
| Ceh2-2022 | 27 | ∞ | [32.6 ; ∞] | 24 | [13 ; 48] |  | 15 | 344.5 | [52.7 ; ∞] | ∞ | [1, ∞] |
| Dau1-2022 | 31 | ∞ | [110.5 ; ∞] | 36 | [21 ; 64] |  | 15 | 209 | [55.5 ; ∞] | 420 | [145, ∞] |
| Deu1-2022 | 28 | 75.8 | [13.7 ; ∞] | 28 | [16 ; 54] |  | 15 | 1870.8 | [62.7 ; ∞] | ∞ | [1, ∞] |
| Deu5-2022 | 23 | 11.5 | [3.1 ; 52.2] | 22 | [12 ; 47] |  | 15 | ∞ | [132.8 ; ∞] | ∞ | [1, ∞] |
| Deu7-2022 | 38 | ∞ | [75.7 ; ∞] | 37 | [23 ; 64] |  | 15 | 21224.7 | [134.4 ; ∞] | ∞ | [1, ∞] |
| Deu8-2022 | 22 | 89.5 | [11.2 ; ∞] | 28 | [16 ; 58] |  | 15 | 1240.4 | [96 ; ∞] | ∞ | [1, ∞] |
| Eng1-2022 | 25 | ∞ | [28.5 ; ∞] | 35 | [20 ; 69] |  | 11 | ∞ | [35.8 ; ∞] | ∞ | [1, ∞] |
| Fri1-2022 | 29 | ∞ | [75.4 ; ∞] | 31 | [18 ; 59] |  | *NA* | *NA* | *NA* | *NA* | *NA* |
| Fri2-2022 | 26 | 82.3 | [23.9 ; ∞] | 27 | [16 ; 49] |  | *NA* | *NA* | *NA* | *NA* | *NA* |
| Ehn1-2021 | 20 | 50.9 | [15.2 ; ∞] | 35 | [18 ; 78] |  | 11 | ∞ | [15.7 ; ∞] | 220 | [67, ∞] |
| Ger1-2021 | 20 | ∞ | [41.2 ; ∞] | 25 | [14 ; 50] |  | 13 | 256.1 | [16.2 ; ∞] | 312 | [101, ∞] |
| Ger3-2021 | 21 | ∞ | [21.3 ; ∞] | 38 | [21 ; 79] |  | 15 | ∞ | [44.4 ; ∞] | ∞ | [1, ∞] |
| Ger4-2021 | 29 | ∞ | [32.9 ; ∞] | 23 | [13 ; 44] |  | 15 | 792.2 | [61.1 ; ∞] | ∞ | [1, ∞] |
| Ger6-2021 | 20 | ∞ | [21.1 ; ∞] | 28 | [16 ; 59] |  | 15 | 724.2 | [130.2 ; ∞] | ∞ | [1, ∞] |
| Gox1-2022 | 27 | 130.3 | [13.4 ; ∞] | 25 | [15 ; 46] |  | *NA* | *NA* | *NA* | *NA* | *NA* |
| Han1-2021 | 25 | 64.7 | [10.9 ; ∞] | 24 | [14 ; 46] |  | 15 | 128.5 | [24.8 ; ∞] | 210 | [86, ∞] |
| Ich0-2022 | 10 | 71 | [6.1 ; ∞] | 30 | [12 ; 613] |  | *NA* | *NA* | *NA* | *NA* | *NA* |
| Jeff1-2022 | 21 | 62.8 | [9.6 ; ∞] | 40 | [23 ; 88] |  | *NA* | *NA* | *NA* | *NA* | *NA* |
| Kes1-2022 | 29 | 139.2 | [28.3 ; ∞] | 32 | [19 ; 59] |  | *NA* | *NA* | *NA* | *NA* | *NA* |
| Koh1-2022 | 34 | 31.4 | [11.3 ; 661.8] | 33 | [20 ; 56] |  | *NA* | *NA* | *NA* | *NA* | *NA* |
| Lac1-2022 | 19 | ∞ | [15.4 ; ∞] | 34 | [19 ; 75] |  | *NA* | *NA* | *NA* | *NA* | *NA* |
| Lan1-2022 | 34 | 691 | [30.8 ; ∞] | 26 | [15 ; 46] |  | *NA* | *NA* | *NA* | *NA* | *NA* |
| Rla3-2021 | 20 | 108 | [13.7 ; ∞] | 20 | [10 ; 41] |  | 17 | 98.5 | [13.6 ; ∞] | 272 | [111, ∞] |
| Rla5-2022 | 27 | 169.6 | [23.5 ; ∞] | 31 | [18 ; 59] |  | *NA* | *NA* | *NA* | *NA* | *NA* |
| Rla6-2021 | 29 | ∞ | [16.8 ; ∞] | 34 | [20 ; 59] |  | 12 | ∞ | [2219.4 ; ∞] | ∞ | [1, ∞] |
| Rlei23-2021 | 24 | 47.6 | [10.5 ; ∞] | 31 | [17 ; 63] |  | 15 | ∞ | [51.2 ; ∞] | ∞ | [1, ∞] |
| Rlei45-2022 | 30 | 60 | [18.2 ; ∞] | 26 | [15 ; 50] |  | *NA* | *NA* | *NA* | *NA* | *NA* |
| Rlei6-2021 | 11 | ∞ | [68.8 ; ∞] | 44 | [18 ; 2023] |  | 11 | ∞ | [26.8 ; ∞] | ∞ | [1, ∞] |
| Rlei7-2021 | 13 | ∞ | [3.3 ; ∞] | 35 | [18 ; 124] |  | 12 | ∞ | [46.5 ; ∞] | ∞ | [1, ∞] |
| Rlei8-2021 | 17 | ∞ | [27.2 ; ∞] | 30 | [16 ; 65] |  | 16 | 279.7 | [24.1 ; ∞] | 240 | [91, ∞] |
| RLK1-2021 | 23 | 30.2 | [11.7 ; ∞] | 29 | [16 ; 56] |  | 12 | ∞ | [20.2 ; ∞] | 132 | [52, ∞] |
| RLK2-2021 | 30 | 54.7 | [19.1 ; ∞] | 35 | [ 21 ; 66] |  | 13 | ∞ | [62.9 ; ∞] | ∞ | [1, ∞] |
| RLK3-2022 | 15 | 191.9 | [8 ; ∞] | 38 | [20 ; 88] |  | *NA* | *NA* | *NA* | *NA* | *NA* |
| RLK4-2022 | 23 | 3520.7 | [9.6 ; ∞] | 27 | [14 ; 53] |  | *NA* | *NA* | *NA* | *NA* | *NA* |
| RLK56-2022 | 18 | 13.3 | [2.3 ; ∞] | 26 | [14 ; 56] |  | *NA* | *NA* | *NA* | *NA* | *NA* |
| Rlmu2-2022 | 20 | 67.4 | [7.3 ; ∞] | 24 | [12 ; 48] |  | *NA* | *NA* | *NA* | *NA* | *NA* |
| Rsc1-2021 | 20 | 367.3 | [14.1 ; ∞] | 23 | [13 ; 49] |  | 15 | 104.8 | [24.4 ; ∞] | 210 | [86, ∞] |
| Rut2-2021 | 27 | ∞ | [43.7 ; ∞] | 31 | [18 ; 57] |  | 15 | ∞ | [18.1 ; ∞] | ∞ | [1, ∞] |
| Rut3-2022 | 23 | ∞ | [28.7 ; ∞] | 27 | [14 ; 51] |  | *NA* | *NA* | *NA* | *NA* | *NA* |
| S2-2022 | 26 | ∞ | [21.2 ; ∞] | 34 | [19 ; 66] |  | *NA* | *NA* | *NA* | *NA* | *NA* |
| S3-2021 | 34 | ∞ | [46.8 ; ∞] | 33 | [20 ; 56] |  | 15 | 8293.4 | [27 ; ∞] | ∞ | [1, ∞] |
| S6-2021 | 31 | 65.3 | [10.3 ; ∞] | 33 | [19 ; 58] |  | 15 | 220.5 | [24.6 ; ∞] | 210 | [85, ∞] |
| S67-2021 | 18 | 141 | [13 ; ∞] | 27 | [14 ; 68] |  | 13 | 110 | [25.1 ; ∞] | 156 | [61, ∞] |
| S17-2021 | 21 | 75.3 | [18.6 ; ∞] | 23 | [13 ; 46] |  | 15 | 377.6 | [65.7 ; ∞] | ∞ | [1, ∞] |
| S21-2021 | 25 | 50.7 | [12.4 ; ∞] | 20 | [10 ; 42] |  | 15 | 41.5 | [15.8 ; ∞] | 52 | [27, 184] |
| V1-2021 | 35 | 57.5 | [19.9 ; ∞] | 29 | [17 ; 51] |  | 15 | 2316.1 | [74.9 ; ∞] | ∞ | [1, ∞] |
| V2-2021 | 29 | 51.8 | [13.3 ; ∞] | 24 | [14 ; 46] |  | 15 | 1658.8 | [88.2 ; ∞] | ∞ | [1, ∞] |
| V3-2021 | 20 | 96.8 | [8 ; ∞] | 35 | [19 ; 73] |  | 15 | 744.5 | [56.2 ; ∞] | 420 | [145, ∞] |
| V4-2021 | 21 | ∞ | [37.9 ; ∞] | 34 | [20 ; 65] |  | 15 | 792.6 | [114.1 ; ∞] | ∞ | [1, ∞] |
| Wei1-2021 | 25 | ∞ | [18.4 ; ∞] | 29 | [17 ; 52] |  | 15 | ∞ | [29.6 ; ∞] | ∞ | [1, ∞] |
| Wur1-2022 | 32 | ∞ | [29.5 ; ∞] | 36 | [22 ; 64] |  | *NA* | *NA* | *NA* | *NA* | *NA* |
| Zp0-2022 | 35 | ∞ | [95.4 ; ∞] | 34 | [21 ; 59] |  | *NA* | *NA* | *NA* | *NA* | *NA* |
| Zp1-2022 | 30 | ∞ | [68.6 ; ∞] | 24 | [14 ; 46] |  | *NA* | *NA* | *NA* | *NA* | *NA* |
| Zp2-2022 | 33 | 18.8 | [4.7 ; 166.7] | 26 | [15 ; 47] |  | *NA* | *NA* | *NA* | *NA* | *NA* |

Table S5**:** *N_e_* estimates from temporal samples, using Pollak’s (1983) estimator (Microsatellites and SNPs), Nei & Tajima’s (1981) estimator (Microsatellites and SNPs), Jorde & Ryman’s (2007) estimator (Microsatellites and SNPs). Values in square brackets represent 95% confidence intervals based on a jackknife procedure.

|  | Sampling Sites | | | | Pollak (1983) | | | | Nei & Tajima (1981) | | | | Jorde & Ryman (2007) | | | |  |
| --- | --- | --- | --- | --- | --- | --- | --- | --- | --- | --- | --- | --- | --- | --- | --- | --- | --- |
|  | Microsatellites | | SNPs | | Microsatellites | | SNPs | | Microsatellites | | SNPs | | Microsatellites | | SNPs | |  |
| Pop | Sample Size 2021 | Sample Size 2022 | Sample Size 2021 | Sample Size 2022 | Estimated *N_e_* | 95% CI | Estimated *N_e_* | 95% CI | Estimated *N_e_* | 95% CI | Estimated *N_e_* | 95% CI | Estimated *N_e_* | 95% CI | Estimated *N_e_* | 95% CI | |
| A5 | 26 | 13 | *ND* | *ND* | -88.7 | [28.6; ∞] |  |  | -215.8 | [18.2; ∞] |  |  | 52.7 | [14; ∞] |  |  | |
| A6 | 30 | 26 | 15 | 14 | -320.5 | [26.1; ∞] | 94.8 | [40.9; ∞] | -125.5 | [30; ∞] | -562.6 | [76.3; ∞] | -94.9 | [27.9; ∞] | 63.4 | [28.0; ∞] | |
| A7 | 33 | 29 | 15 | 15 | 1387.9 | [25.2; ∞] | 4559 | [89.9; ∞] | -500.3 | [30.6; ∞] | -241.3 | [145.5; ∞] | -274.4 | [46.3; ∞] | 422.9 | [62.6; ∞] | |
| A8 | 30 | 36 | 15 | 15 | 16.1 | [1.9; ∞] | 297 | [69.3; ∞] | 21.3 | [2.6; ∞] | -137.5 | [312.4; ∞] | 25.1 | [7.3; ∞] | -273.1 | [115.8; ∞] | |
| A9 | 31 | 27 | 13 | 15 | 90.5 | [8.6; ∞] | 416.7 | [64.4; ∞] | 59.2 | [7.3; ∞] | -121.5 | [204.6; ∞] | 21.7 | [6.6; ∞] | -837 | [70.8; ∞] | |
| A11 | 31 | 39 | 13 | 15 | -58.1 | [189.6; ∞] | 89.3 | [40.4; ∞] | -55.8 | [400.7; ∞] | -243.2 | [99.3; ∞] | -63.3 | [2284.4; ∞] | 98.3 | [34.4; ∞] | |
| A14 | 27 | 23 | 14 | 15 | -91.9 | [23.5; ∞] | 43 | [26.8; 94.7] | -51.2 | [48.4; ∞] | 80.4 | [37.4; ∞] | -29.7 | [70.9; ∞] | 38.8 | [23.0; 124.5] | |
| A16 | 30 | 34 | 15 | 15 | 344.8 | [14; ∞] | 110.7 | [79.8; ∞] | 315.4 | [13.4; ∞] | 1011 | [79.8; ∞] | 360.9 | [18.7; ∞] | 89.3 | [39.5; ∞] | |
| CB4 | 18 | 21 | 14 | 15 | 116 | [19; ∞] | 492.4 | [64.2; ∞] | 99.1 | [14.7; ∞] | -140.2 | [131.4; ∞] | 75.4 | [13.5; ∞] | 140.9 | [40.6; ∞] | |
| Ceh1 | 31 | 13 | 11 | 12 | -33.9 | [16.5; ∞] | 51.4 | [29.0; 170.3] | -33.5 | [17.8; ∞] | 174.8 | [51.3; ∞] | -62.4 | [21.5; ∞] | 48.8 | [26.1; 369.6] | |
| Ceh2 | 25 | 27 | 12 | 15 | 55.3 | [12.9; ∞] | 76 | [37.3; 1192.0] | 75.6 | [13.7; ∞] | 319 | [61.2; ∞] | 104 | [16.9; ∞] | 77.2 | [34.6; ∞] | |
| Dau1 | 26 | 31 | 15 | 15 | 61.3 | [12.5; ∞] | 85.3 | [42.5; 945.0] | 44.3 | [11.9; ∞] | 280.1 | [67.4; ∞] | 23.1 | [9.6; ∞] | 93.3 | [41.5; ∞] | |
| Ger1 | 20 | 33 | *NA* | *NA* | 97.9 | [10.2; ∞] | *NA* | *NA* | 74.4 | [7.3; ∞] | *NA* | *NA* | 38.3 | [7.8; ∞] | *NA* | *NA* | |
| Ger3 | 21 | 12 | *NA* | *NA* | -48.6 | [14.6; ∞] | *NA* | *NA* | -35.3 | [22.3; ∞] | *NA* | *NA* | -26.8 | [57.1; ∞] | *NA* | *NA* | |
| Ger4 | 29 | 30 | *NA* | *NA* | -104.8 | [32.1; ∞] | *NA* | *NA* | -199.7 | [25.1; ∞] | *NA* | *NA* | 1144.6 | [30.4; ∞] | *NA* | *NA* | |
| Rla5 | 21 | 27 | *NA* | *NA* | -184.5 | [19.1; ∞] | *NA* | *NA* | -132.1 | [20; ∞] | *NA* | *NA* | -115.8 | [26.8; ∞] | *NA* | *NA* | |
| Rla6 | 29 | 28 | *NA* | *NA* | 57.4 | [15; ∞] | *NA* | *NA* | 51.2 | [13; ∞] | *NA* | *NA* | 51.4 | [15.8; ∞] | *NA* | *NA* | |
| Rlei23 | 24 | 15 | *NA* | *NA* | -77 | [14.6; ∞] | *NA* | *NA* | -55.5 | [22.2; ∞] | *NA* | *NA* | -41.3 | [60.7; ∞] | *NA* | *NA* | |
| Rlei6 | 11 | 29 | *NA* | *NA* | -236 | [26.1; ∞] | *NA* | *NA* | -140.7 | [29.6; ∞] | *NA* | *NA* | -339.7 | [24.9; ∞] | *NA* | *NA* | |
| Rlei7 | 13 | 34 | *NA* | *NA* | -53.3 | [21.4; ∞] | *NA* | *NA* | -35.8 | [31.1; ∞] | *NA* | *NA* | -28.6 | [120.6; ∞] | *NA* | *NA* | |
| Rlei8 | 17 | 26 | *NA* | *NA* | 201.5 | [11.1; ∞] | *NA* | *NA* | -1023.5 | [13.4; ∞] | *NA* | *NA* | 81.3 | [14.3; ∞] | *NA* | *NA* | |
| RLK1 | 23 | 18 | *NA* | *NA* | 82.1 | [9.3; ∞] | *NA* | *NA* | 242.3 | [12.3; ∞] | *NA* | *NA* | 144.4 | [16.1; ∞] | *NA* | *NA* | |
| RLK2 | 30 | 33 | *NA* | *NA* | 94.1 | [14.2; ∞] | *NA* | *NA* | 177.2 | [14.2; ∞] | *NA* | *NA* | 282.7 | [18.2; ∞] | *NA* | *NA* | |
| Rsc1 | 20 | 20 | *NA* | *NA* | 21.4 | [7.6; ∞] | *NA* | *NA* | 21.2 | [8.1; ∞] | *NA* | *NA* | 16.4 | [8.5; ∞] | *NA* | *NA* | |
| Rut2 | 27 | 28 | *NA* | *NA* | 52 | [12.4; ∞] | *NA* | *NA* | 164.5 | [18.6; ∞] | *NA* | *NA* | -231.1 | [38; ∞] | *NA* | *NA* | |
| S3 | 34 | 20 | *NA* | *NA* | -55.7 | [120.2; ∞] | *NA* | *NA* | -62.4 | [72.3; ∞] | *NA* | *NA* | -52.7 | [132.3; ∞] | *NA* | *NA* | |
| S6 | 31 | 23 | *NA* | *NA* | 151.4 | [15.9; ∞] | *NA* | *NA* | 64.4 | [12; ∞] | *NA* | *NA* | 42.9 | [12.7; ∞] | *NA* | *NA* | |
| S67 | 18 | 20 | *NA* | *NA* | 33.3 | [10.2; ∞] | *NA* | *NA* | 40.5 | [11.7; ∞] | *NA* | *NA* | 45.6 | [11.3; ∞] | *NA* | *NA* | |
| S21 | 25 | 11 | *NA* | *NA* | 12 | [4.4; 84.9] | *NA* | *NA* | 14.1 | [4.1; ∞] | *NA* | *NA* | 11 | [4; ∞] | *NA* | *NA* | |
| V1 | 35 | 33 | *NA* | *NA* | -107.3 | [60.2; ∞] | *NA* | *NA* | -80 | [85.1; ∞] | *NA* | *NA* | -59.4 | [343.5; ∞] | *NA* | *NA* | |
| V2 | 29 | 30 | *NA* | *NA* | 21.5 | [7.4; 173.8] | *NA* | *NA* | 16.8 | [5.8; 89.1] | *NA* | *NA* | 10.3 | [4.7; ∞] | *NA* | *NA* | |
| V3 | 20 | 33 | *NA* | *NA* | -40.4 | [67.5; ∞] | *NA* | *NA* | -46.8 | [31.2; ∞] | *NA* | *NA* | -62.3 | [33.9; ∞] | *NA* | *NA* | |
| V4 | 21 | 34 | *NA* | *NA* | 50.2 | [11.8; ∞] | *NA* | *NA* | 60.1 | [13.3; ∞] | *NA* | *NA* | 67.4 | [17.1; ∞] | *NA* | *NA* | |
| Wei1 | 25 | 27 | *NA* | *NA* | 260.6 | [33.3; ∞] | *NA* | *NA* | -1039.8 | [29.9; ∞] | *NA* | *NA* | 178.6 | [23.2; ∞] | *NA* | *NA* | |

Table S6: **(a)** Metapopulation *N_e_*_Bayesian clustering (identical to table 2a) estimates and **(b)** Metapopulation *N_e_*_Bayesian clustering estimates taking account for the potential migrants (*i.e.* individuals displaying a proportion of admixture > 20 % were removed from the analyses) as detected in FastStructure for *K* = 3 in both the North (GA) and South (GB) regions. Single-sample *N*_e_ were estimated for the SNP dataset using the Linkage Disequilibrium method (Hill, 1981; Waples, 2006; Waples and Do, 2010) and sibship assignment (Wang., 2009). Values in square brackets represent 95% confidence intervals based on a jackknife procedure.

|  | 1. Metapopulation *N*_e__Bayesian clustering | | | | |  | 1. Metapopulation *N*_e__Bayesian clustering (no admixed individuals) | | | | |
| --- | --- | --- | --- | --- | --- | --- | --- | --- | --- | --- | --- |
|  | Sample size | LD method | | Sibship assignment method | |  | Sample size | LD method | | Sibship assignment method | |
| Group |  | Estimated *Ne* | 95% CI | Estimated *Ne* | 95% CI |  |  | Estimated *Ne* | 95% CI | Estimated *Ne* | 95% CI |
| GA-1 | 222 | 938.8 | [659.5 ; 1650.5] | 4673 | [3246 ; 8305] |  | 140 | 835.3 | [474.9 ; 3260.6] | 2780 | [1799 ; 5945] |
| GA-2 | 181 | 771.4 | [499.6 ; 1686.2] | 2962 | [2026 ; 5206] |  | 85 | 617.5 | [259.9 ; ∞] | 1785 | [1078 ; 4943] |
| GB-1 | 30 | 150.0 | [59.5 ; ∞] | 218 | [127 ; 582] |  | 4 | NA | NA | NA | NA |
| GB-2 | 297 | 954.0 | [796.8 ; 1220.9] | 3822 | [2999 ; 5409] |  | 144 | 1055.6 | [700.4 ; 2412.8] | 10296 | [5047 ; 249085] |
| GB-3 | 60 | 1669.9 | [594.7 ; ∞] | ∞ | [1 ; ∞] |  | 20 | 12648.9 | [117.2 ; ∞] | ∞ | [1 ; ∞] |
